# Global incidence of suicide among Indigenous peoples: a systematic review

**DOI:** 10.1186/s12916-018-1115-6

**Published:** 2018-08-20

**Authors:** Nathaniel J. Pollock, Kiyuri Naicker, Alex Loro, Shree Mulay, Ian Colman

**Affiliations:** 1 Division of Community Health and Humanities, Faculty of Medicine, Memorial University, Prince Philip Drive, St. John’s, Newfoundland and Labrador A1B 3V6 Canada; 2 Labrador Institute of Memorial University, P.O. Box 490, Stn. B, 219 Hamilton River Road, Happy Valley-Goose Bay, ,Newfoundland and Labrador A0P 1E0 Canada; 3 School of Epidemiology and Public Health, Faculty of Medicine, University of Ottawa, 600 Peter Morand Cr, Room 308C, Ottawa, ON K1G 5Z3 Canada

**Keywords:** Indigenous, First peoples, Inuit, Health disparities, Suicide, Mortality, Surveillance, Epidemiology

## Abstract

**Background:**

Suicide is the second leading cause of death among adolescents worldwide, and is a major driver of health inequity among Indigenous people in high-income countries. However, little is known about the burden of suicide among Indigenous populations in low- and middle-income nations, and no synthesis of the global data is currently available. Our objective was to examine the global incidence of suicide among Indigenous peoples and assess disparities through comparisons with non-Indigenous populations.

**Methods:**

We conducted a systematic review of suicide rates among Indigenous peoples worldwide and assessed disparities between Indigenous and non-Indigenous populations. We performed text word and Medical Subject Headings searches in PubMed, MEDLINE, Embase, Cumulative Index of Nursing and Allied Health (CINAHL), PsycINFO, Latin American and Caribbean Health Sciences Literature (LILACS), and Scientific Electronic Library Online (SciELO) for observational studies in any language, indexed from database inception until June 1, 2017. Eligible studies examined crude or standardized suicide rates in Indigenous populations at national, regional, or local levels, and examined rate ratios for comparisons to non-Indigenous populations.

**Results:**

The search identified 13,736 papers and we included 99. Eligible studies examined suicide rates among Indigenous peoples in 30 countries and territories, though the majority focused on populations in high-income nations. Results showed that suicide rates are elevated in many Indigenous populations worldwide, though rate variation is common, and suicide incidence ranges from 0 to 187.5 suicide deaths per 100,000 population. We found evidence of suicide rate parity between Indigenous and non-Indigenous populations in some contexts, while elsewhere rates were more than 20 times higher among Indigenous peoples.

**Conclusions:**

This review showed that suicide rates in Indigenous populations vary globally, and that suicide rate disparities between Indigenous and non-Indigenous populations are substantial in some settings but not universal. Including Indigenous identifiers and disaggregating national suicide mortality data by geography and ethnicity will improve the quality and relevance of evidence that informs community, clinical, and public health practice in Indigenous suicide prevention.

**Electronic supplementary material:**

The online version of this article (10.1186/s12916-018-1115-6) contains supplementary material, which is available to authorized users.

## Background

Globally, suicide accounts for approximately 800,000 deaths annually [1] and is the second leading cause of mortality among adolescents [2]. According to the World Health Organization (WHO), low- and middle-income countries and high-income countries have similar annual age-standardized suicide rates at 11.2 and 12.7 per 100,000 respectively; however, low- and middle-income countries account for 75% of suicide deaths worldwide [1]. National suicide rates range from less than one to 44 per 100,000 population, though there is often a disproportionate burden among specific subgroups within countries, such as Indigenous peoples [1]. Studies from high-income countries including Australia [3, 4], New Zealand [5], the USA [6, 7], Canada [8–10], and other Arctic nations [11–14] consistently find elevated suicide rates among Indigenous populations, with substantial rate disparities compared to non-Indigenous populations. Several studies have shown that regional suicide rates vary greatly among Indigenous peoples, and that some Indigenous populations have low rates or no incidence of suicide [15, 16].

Indigenous peoples and nations differ vastly in culture, language, political autonomy, and relative wealth, yet many face similar social disadvantages and health disparities as a result of colonization [17–19]. Colonial governments have used discriminatory legislation and policies to deny rights and economic opportunities, and have attempted to acculturate Indigenous people into non-Indigenous societies [17, 19, 20]. Structural violence meted out by governments has taken many forms including dispossessing Indigenous peoples from traditional and sovereign lands, forced settlement and relocation, and outlawing cultural practices and languages [17–21]. This violence is grossly evident in the twentieth century assimilationist policies of former British colonies such as Canada and Australia. Indigenous children were systematically removed from their communities and placed in non-Indigenous institutions or families with the policy mandate to “weaken family ties and cultural linkages, and to indoctrinate children into a new culture” ([20], p. v). The contemporary legacy of this type of social engineering manifests in differential exposures to health threats and in inequitable outcomes that show up across generations [20, 22]. Intergenerational trauma from institutionalized abuse and racism experienced by Indigenous peoples has been linked to persistent social and mental health problems in some communities [19, 20, 23].

Although evidence has shown a disproportionate burden of suicide among Indigenous populations in national and regional studies, a global and systematic investigation of this topic has not been undertaken to date. Previous reviews of suicide epidemiology among Indigenous populations have tended to be less comprehensive or not systematic, and have often focused on subpopulations such as youth [24, 25], high-income countries [9, 26], or regions such as Oceania [27] or the Arctic [24, 28]. Given that approximately 80% of the world’s more than 300 million Indigenous people live in Asia, Latin America, and Africa [17, 18], a comprehensive study of global suicide rates that includes low- and middle-income countries is needed. Our aim was to examine the published findings on the incidence of suicide among Indigenous peoples worldwide, and to compare rates with non-Indigenous or general populations to assess relative disparities.

## Methods

### Search strategy

We systematically reviewed findings on the incidence of suicide in Indigenous populations worldwide. We searched for studies that analyzed population-based data on suicide deaths, and included papers that reported crude or standardized mortality rates. Health science librarians were consulted about the design of the search strategy with the aim to capture all peer-reviewed literature. The search combined terms related to three concept areas: population (Indigenous), outcome (suicide mortality rates), and study design (observational). Term selection was based on previous systematic reviews and combined key terms adapted for each database and also Medical Subject Headings (MeSH) as applicable. The study protocol is available in Additional file 1: Supplement 1. Additional details about the methods are reported in Additional file 1: Supplement 2, including citations for previous reviews, a list of included terms, a description of the procedures used for study selection and eligibility criteria, and a complete list of databases and hand-searched review articles.

One author (NJP) performed online text word and MeSH searches for articles indexed in PubMed, MEDLINE, Embase, Cumulative Index of Nursing and Allied Health (CINAHL), PsycINFO, Latin American and Caribbean Health Sciences Literature (LILACS), and Scientific Electronic Library Online (SCiELO). A second author (KN) replicated the search in PubMed and obtained the same number of articles as the first author. We searched for studies in any language, indexed from database inception until June 1, 2017. We conducted a secondary search with a comprehensive list of terms for specific tribal groups, nations, and populations identified in previous reviews. As no additional studies were identified, this approach validated the primary search. We also searched the WHO’s regional medical literature indexes, Indigenous-specific online research portals, and journals focused on Indigenous health. We hand-searched the reference lists of included articles and previous reviews to identify other eligible studies. Additional file 1: Supplement 2 includes a list of all databases and hand-searched sources.

One author (NJP) imported the results into a reference management program and removed duplicates. Two authors (NJP and KN) read the abstracts and screened in papers if they (1) reported a population-based crude and/or standardized suicide rate, or count and population data; (2) reported a rate for an Indigenous population; and (3) used an observational design. We excluded articles that did not include an Indigenous population, focused only on a specific age, gender, clinical subgroup, or deaths from a specific cause (for example, firearms), or were not peer-reviewed. Articles were also excluded if they were iterations, program evaluations or experimental studies, not primary studies, from the gray literature, or used identical data sources as prior studies.

Although there is no international consensus on the definition of Indigenous, we used the United Nation’s working definition to assess study population eligibility [17, 18]. The UN's conceptualization of Indigenous involves self and group identification; a special attachment to and use of traditional land, distinct knowledge, language, and culture; distinct social, economic, and political systems; common ancestry with original territorial occupants; participation in maintenance and reproduction of distinct ethnic identity; and a non-dominant socio-political status [17, 18]. A paper was eligible based on this criterion if it reported an outcome for an Indigenous population, tribe, community, nation, or group, including papers that used the geographic proxy method. For the proxy method, census data is used to detect areas where Indigenous people are a majority population [29, 30]. We considered an area to be a proxy identifier if 80% or more of the population self-identified as Indigenous.

Two authors reviewed the full text of each paper and assessed eligibility based on inclusion criteria. At this stage, we excluded papers that did not report rates for the majority of the population (aged 15–65 years), did not conduct the primary data analysis, or provided rates in figures only and did not report count and population data. If two eligible articles used the same data source with a period of overlap, we included the article with the longer study period. During screening, full text review, and data extraction, we resolved disagreements through discussion or consultation with a third author. Translators helped assess non-English language articles and assisted with data extraction for four included studies. The following data was independently extracted by two authors (AL and NJP), then compared: citation, study design, country and region/community, Indigenous population, data source, standard population, number of suicide deaths, population count, crude and standardized suicide rates (overall and by gender and age group), comparative rates for a non-Indigenous or general population, and the measure of relative effect (incidence rate ratio).

### Data analysis

We summarized all included studies in a table and reported counts, population, crude and standardized suicide mortality rates, and rate ratios. We calculated crude suicide mortality incidence rates for articles that reported only count and population data, and we estimated rate ratios when not otherwise reported by dividing the Indigenous population rate by the comparison population rate. To identify global patterns, we presented rates and rate ratios in tables and figures grouped by WHO region, country, population, and gender; we did not pool the data due to heterogeneity. We also reported on trends in suicide mortality over time and by age group; reported time trends reflect results from included studies, not pooled and recalculated rates. We modified the Newcastle-Ottawa Scale and used it to assess the quality of included articles. Additional file 1: Supplement 2 includes a description of the quality assessment procedures and scoring, and the Preferred Reporting Items for Systematic Reviews and Meta-Analyses (PRISMA) checklist is provided in Additional file 1: Supplement 4 [31].

## Results

**Fig. 1:**
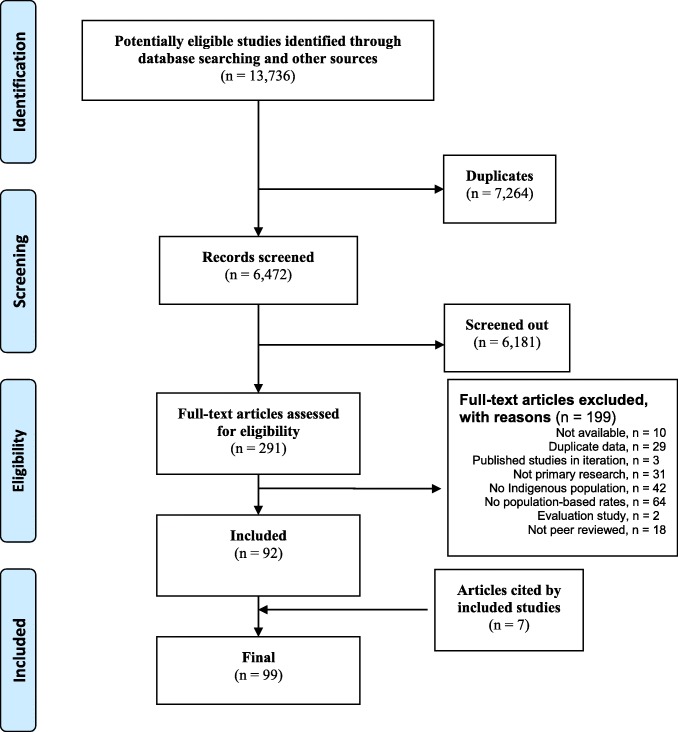
Flow diagram of study selection

The search identified 13,736 papers; after removing duplicates, screening abstracts, and full text review, we included 99 in our analysis (Fig. 1). Included studies examined suicide rates in Indigenous populations in 30 countries and territories across six decades (Table 1), though the majority focused on those in high-income countries such as American Indian and Alaska Natives in the USA (*n* = 35) and Inuit and First Nations in Canada (*n* = 14). Studies in low- and middle-income countries (*n* = 22) were mostly from Brazil (*n* = 4), China and Taiwan (*n* = 6), and Fiji (*n* = 5). Coverage included circumpolar Indigenous peoples such as Sámi (*n* = 3) and Nenets (*n* = 1), and populations from the Western Pacific region including Aboriginal and Torres Strait Islanders in Australia (*n* = 6) and Māori and other Pacific peoples (*n* = 16). Four studies were transnational comparisons [32–35], though numerous papers included multiple Indigenous groups within a single country. Studies were mostly of moderate quality (mean 2.79 on a 4-point scale) based on our assessment of study characteristics, as reported in Additional file 1: Supplement 3, Tables S1 and S2.

**Table 1:** Overview of included studies

	No. of studies (*N*)	% of total (*n*/99)
Decade of publication		
1960–1979	12	12.1%
1980s	23	23.2%
1990s	25	25.3%
2000s	17	17.2%
2010s	22	22.2%
World Bank income		
High-income	76	76.8%
Low- and middle-income	22	22.2%
Multiple	1	1.0%
WHO Region		
Western Pacific	33	33.3%
European	8	8.1%
Region of the Americas	56	56.6%
Multiple regions	2	2.0%
Total Indigenous population		
Less than 10,000	17	17.2%
10,000–99,999	32	32.3%
100,000–999,999	12	12.1%
1,000,000+	4	4.0%
Not reported	34	34.3%
Number of suicide deaths among Indigenous population		
Less than 20	18	18.2%
21–99	23	23.2%
100–999	23	23.2%
1000+	4	4.0%
Not reported	31	31.3%

### Incidence

We extracted population-based suicide mortality rates from 93 papers (Table 2) and included gender-specific incidence data from six additional studies [5, 10, 36–39]. Overall, suicide rates among Indigenous peoples varied at all levels of aggregation in both high-income and low- and middle-income countries, and spanned from zero to 187.5 deaths per 100,000 person-years (PY; Table 2). In high-income countries, national and provincial suicide rates among Indigenous peoples ranged from 1.7 per 100,000 in Brunei Darussalam [40] to 50.4 per 100,000 among Aboriginal and Torres Strait Islanders in Northern Territory, Australia [41]. Rates in high-income countries were highest among rural Indigenous populations and in sparsely populated regions such as the Arctic. Among low- and middle-income countries, Palawan communities in the Philippines had the highest crude suicide rates (134 per 100,000) [42], while Indigenous peoples in Malaysia [43] and some Pacific small island states such as Fiji had crude rates under 7 per 100,000 population. The number of suicide deaths used for rate calculations ranged from zero to 4219 (Table 2).

**Table 2:** Suicide mortality rates among Indigenous populations by WHO region and country

WHO Region	Country	Indigenous peoples^a^	Population	Period	Deaths	CSIR	SSIR
European Region							
Soininen (2008) [14]	Finland (Northern region)	Sámi	2091	1979–2005	24	50.0	–
Thorslund (1989) [70]	Greenland	Kalaallit (Inuit)	–	1986	57	129	–
Bjerregaard (2015) [12]	Greenland	Kalaallit (Inuit)	57,000	1970–2011	1678	87.7	–
	East/north regions	Kalaallit (Inuit)	–	1970–2011	–307	187.5	–
	Nuuk	Kalaallit (Inuit)	–	1970–2011	–303	86.6	–
	Towns in Western region	Kalaallit (Inuit)	–	1970–2011	–837	81.2	–
	Villages in Western region	Kalaallit (Inuit)	–	1970–2011	–222	61.4	–
Klomek (2016) [71]	Israel	Bedouin	–	1999–2011	39	4.4	3.2
Silviken (2009) [11]	Norway (Northern region)	Sámi	19,801	1970–1998	89	18.9	–
Sumarokov (2014) [72]	Russia (Nenets Autonomous Okrug)	Nenets	7504	2002–2012	67	79.8	72.7
Hassler (2005) [13]	Sweden	Sámi	41,721	1961–2000	114	11.7	–
		Sámi (non-herding)	–	1961–2000	76	9.8	–
		Sámi (reindeer herding)	–	1961–2000	38	19.6	–
Western Pacific Region (Australia)							
Clayer (1991) [73]	Australia (South Australia)	Aboriginal and Torres Strait Islander	13,298	1988	14	105.3	–
Cantor (1997) [74]	Australia (Queensland)	Aboriginal and Torres Strait Islander	–	1990–1992	–	–	17.1
Stevenson (1998) [34]	Australia	Aboriginal and Torres Strait Islander	–	1990–1992	67	–	11.1
Bramley (2004) [32]	Australia	Aboriginal and Torres Strait Islander	–	1999	–	–	19.4
De Leo (2011) [4]	Australia (Queensland)	Aboriginal and Torres Strait Islander	–	1994–2007	544	–	27.2
Measey (2006) [41]	Australia (Northern Territory)	Aboriginal and Torres Strait Islander	–	2002	–	–	50.4
Pridmore (2009) [3]	Australia (Northern Territory)	Aboriginal and Torres Strait Islander	–	2001–2006	130	–	36.7
Campbell (2016) [75]	Australia (Kimberley)	Aboriginal and Torres Strait Islander	11,550	2005–2014	102	–	74
Western Pacific Region (Oceania)							
Booth (1999) [33]	American Samoa	Samoan	54,800	1990–1991	–	18	–
Hezel (1984) [76]	FSM (Chuuk)	Chuukese	37,488	1971–1983	129	30	–
Hezel (1989) [35]	Federated States of Micronesia	Pacific peoples	142,298	1984–1987	134	25.8	–
	Chuuk	Chuukese	44,000	1984–1987	51	28.3	–
	Kosrae	Kosraen	6448	1984–1987	6	25.9	–
	Pohnpei	Pohnpeian	28,879	1984–1987	18	16.7	–
	Yap	Yapese	10,139	1984–1987	5	20.2	–
Booth (1999) [33]	Federated States of Micronesia	Pacific peoples	105,700	1988–1992	–	31	–
	Chuuk	Chuukese	–	1988–1992	–	35	–
	Kosrae	Kosraen	–	1988–1992	–	48	–
	Pohnpei	Pohnpeian	–	1988–1992	–	20	–
	Yap	Yapese	–	1988–1992	–	48	–
Ree (1971) [77]	Fiji (Macuata)	iTaukei	9950	1962–1968	4	5.7	–
Price (1975) [51]	Fiji	iTaukei	–	1971–1972	6	1.3	–
Haynes (1984) [78]	Fiji (Macuata)	iTaukei	8111	1979–1982	2	6.7	–
Pridmore (1994) [79]	Fiji (Western Division)	iTaukei	–	1986–1992	–	2	–
Pridmore (1995) [80]	Fiji	iTaukei	–	1969–1989	–	3.6	–
Booth (1999) [33]	Fiji	iTaukei	–	1982–1983	–	3	3
Booth (1999) [33]	French Polynesia	Polynesian	218,000	1988–1992	–	9	9
Booth (2010) [81]	Guam	Chamorro	–	1998–2000	–	21	–
Hezel (1989) [35]	Marshall Islands	Marshallese	39,060	1984–1987	39	26.5	–
Booth (1999) [33]	Marshall Islands	Marshallese	54,700	1988–1992	–	26	–
Langley (1990) [82]	Aotearoa/New Zealand	Māori	–	1984	22	–	8
Langley (2000) [83]	Aotearoa/New Zealand	Māori	–	1985–1994	271	8.8	–
Bramley (2004) [32]	Aotearoa/New Zealand	Māori	–	1999	–	–	12.9
Hezel (1989) [35]	Palau	Palauan	13,772	1984–1987	15	28.8	–
Booth (1999) [33]	Palau	Palauan	16,500	1988–1992	–	29	–
Parker (1966) [84]	Papua New Guinea	Pacific peoples	–	1961–1965	41	0.7	–
Smith (1981) [50]	Papua New Guinea (Southern Highlands)	Huli	50,000	1971–1976	26	17	–
Booth (1999) [33]	Papua New Guinea	Pacific peoples	4,216,100	1990	–	< 1	–
Booth (1999) [33]	Samoa	Samoan	163,400	1981	–	31	34
Pridmore (1997) [49]	Solomon Islands (Honiara area)	Pacific peoples	75,000	1989–1993	13	3.9	–
Vivili (1999) [85]	Tonga	Tongan	98,200	1982–1997	43	2.9	–
Booth (1999) [33]	Vanuatu	ni-Vanuatu	164,100	1990–1992	–	3	–
De Leo (2013) [86]	Vanuatu	ni-Vanuatu	245,619	2010	2	0.8	–
Western Pacific Region (East Asia)							
Telisinghe (2014) [40]	Brunei Darussalam	Indigenous peoples (7 tribes)^b^	14,000	1991–2010	4	1.7	–
Wang (1997) [87]	China (Hohhot, Inner Mongolia)	Meng	27,000	1986–1991	–	2.4	–
		Hui	21,600	1986–1991	–	1.2	–
Lu (2013) [44]	China (Yunnan Province)	Dai	325,126	2004–2005	–	12	–
		Yi	582,596	2004–2005	–	20.8	–
		Li su	147,794	2004–2005	–	50.8	–
		Other ethnic minorities	1,922,430	2004–2005	–	0.96–36.4^c^	
Ali (2014) [43]	Malaysia (Sabah and Sarawak)	Bumiputera	2,981,300	2009	11	0.4	–
Jollant (2014) [42]	Philippines	Palawan	1192	2002–2012	16	134	–
Cheng (1992) [88]	Taiwan	Atayal	–	1981–1985	–	46.3	–
		Ami	–	1981–1985	–	5.3	–
		Bunun	–	1981–1985	–	64.8	–
		Paiwan	–	1981–1985	–	16.3	–
Hsieh (1994) [89]	Taiwan	Indigenous peoples	200,000	1971–1990	1597	40.1	–
		Atayal	–	1971–1990	928	57.6	–
		Bunun	–	1971–1990	222	44.7	–
		Paiwan	–	1971–1990	204	21.3	–
Wen (2004) [90]	Taiwan	Indigenous peoples	200,537	1998–2000	128	21.9	–
Liu (2011) [91]	Taiwan (East region)	Ami	–	1979–1981	30	15.6	–
		Atayal	–	1979–1981	30	68.2	–
Region of the Americas (Brazil and Canada)							
Coloma (2006) [45]	Brazil (Mato Grosso do Sul)	Indigenous peoples (6 tribes)^d^	53,325	2000–2003	194	96.2	–
Souza (2013) [46]	Brazil (Amazonas)	Indigenous peoples	184,764	2006–2010	131	–	18.4
	Manaus	Indigenous peoples	–	2006–2010		–	0
	Sao Gabriel da Cachoeira	Indigenous peoples	–	2006–2010		–	41.9
	Tabatinga	Indigenous peoples	–	2006–2010		–	75.8
Machado (2015) [92]	Brazil	Indigenous peoples	–	2012	–	14.4	–
Orellana (2016) [21]	Brazil (Mato Grosso do Sul)	Indigenous peoples (3 tribes)^e^	75,000	2009–2011	–	–	65.2
Butler (1965) [93]	Canada (NWT/Nunavut)	Inuit	7949	1959–1964	9	18.8	–
	NWT	First Nation	5284	1959–1964	0	0	–
	Yukon	First Nation	2207	1959–1964	5	37.7	–
Young (1983) [94]	Canada (Northwestern Ontario)	Cree and Ojibway	10,000	1972–1981	17	16.1	–
Fox (1984) [95]	Canada (Wikwemikong, Ontario)	Anishinaabe	3000	1976–1980	–	26.7	–
Wotton (1985) [96]	Canada (Labrador)	Innu and Inuit	2500	1979–1983	8	65.5	–
Spaulding (1985) [97]	Canada (Northern Ontario)	Ojibway	3005	1975–1982	14	61.7	–
Mao (1986) [98]	Canada (7 provinces)	First Nation (on reserve)	168,529	1977–1982	344	34	–
Ross (1986) [68]	Canada	Cree	2822	1981–1984	7	83	–
Garro (1988) [99]	Canada (Manitoba)	First Nation (Status Indians)	43,000	1973–1982	174	40.2	–
		Dene	–	1973–1982	–	13	–
		Ojibway (Northern)	–	1973–1982	–	5	–
		Cree	–	1973–1982	–	22	–
		Saulteaux	–	1973–1982	–	48	–
		Dakota	–	1973–1982	–	80	–
Malchy (1997) [100]	Canada (Manitoba)	First Nation and Métis	–	1988–1994	227	38	31.8
Chandler (1998) [16]	Canada (British Colombia)	First Nation	–	1987–1992	220	45.2	–
Isaacs (1998) [101]	Canada (NWT)	Dene	–	1994–1996	–	29	–
	NWT/Nunavut	Inuit	–	1994–1996	–	79	–
Bramley (2004) [32]	Canada	First Nation	–	1999	–	–	27.8
Macaulay (2004) [8]	Canada (Kivalliq, Nunavut)	Inuit	7131	1987–1996	31	–	45.1
Penney (2009) [102]	Canada (Nunavut)	Inuit	20,489	1999–2003	–	–	95.6
	Canada (Nunavik)	Inuit	7628	1999–2003	–	–	159.8
Pollock (2016) [30]	Canada (Labrador)	Innu	1815	1993–2009	28	–137.0	114
		Inuit	2415	1993–2009	64	–186.8	165.6
Region of the Americas (USA, National)							
Ogden (1970) [103]	USA (24 Western states)	American Indian and Alaska Native	630,000	1967	94	17	23.1
Young (1993) [104]	USA (IHSA)	American Indian and Alaska Native	–	1979–1981	–	18.6	–
Lester (1994) [105]	USA	American Indian and Alaska Native	–	1980	–	13.3	–
Lester (1995) [106]	USA (48 states)	American Indian and Alaska Native	984–166,464¶	1980	–	0.0–64.7^f^	
Stevenson (1998) [34]	USA	American Indian	–	1990–1992	572	–	15.5
Bramley (2004) [32]	USA	American Indian and Alaska Native	–	1999	–	–	12
Howard (2014) [107]	USA	American Indian and Alaska Native	2,439,419	1999–2010	4219	–	14.2
Herne (2014) [6]	USA (IHSA)	American Indian and Alaska Native	–	1999–2009	3600	–	21.1
	Pacific Coast IHSA	American Indian and Alaska Native	–	1999–2009	532	–	18.2
	Southwest IHSA	American Indian and Alaska Native	–	1999–2009	1066	–	19.9
	South Plains IHSA	American Indian & Alaska Native	–	1999–2009	626	–	18.7
	North Plains IHSA	American Indian and Alaska Native	–	1999–2009	755	–	26.2
	East IHSA	American Indian and Alaska Native	–	1999–2009	93	–	8.4
	Alaska IHSA	American Indian and Alaska Native	–	1999–2009	528	–	42.5
Region of the Americas (USA, Alaska)							
Kraus (1979) [108]	USA (Alaska)	Alaska Native	56,477	1970	–	29.6	–
Travis (1983) [109]	USA (Alaska)	Alaska Native	–	1975–1979	–	15.8–52.6^g^	
Travis (1984) [110]	USA (NANA, Alaska)	Inupiat	7345	1974–1980	–	106	–
	USA (Arctic Slope, Alaska)	Inupiat	–	1974–1980	–	19.2	–
Hlady (1988) [111]	USA (Alaska)	Alaska Native	–	1983–1984	65	–	42.9
Forbes (1988) [112]	USA (Alaska)	Alaska Native	–	1985	47	64.9	68.8
Kettl (1991) [113]	USA (Alaska)	Alaska Native	–	1979–1984	90	23.4	–
Andon (1997) [114]	USA (Alaska)	Athabascan	6041	1977–1987	40	55.1	–
Marshall (1998) [115]	USA (Alaska)	Alaska Native	25,000	1979–1990	186	49	–
		Yupik	–	1979–1990	103	53	–
		Inupiat	–	1979–1990	60	89	–
		Athabascan	–	1979–1990	23	147	–
Day (2003) [47]	USA (Alaska)	Alaska Native	91,300	1989–1998	–	–	49.7
Day (2009) [116]	USA (Alaska)	Alaska Native	97,012	1999–2003	204	–	36.1
Wexler (2012) [7]	USA (Northwestern Alaska)	Alaska Native	7965	2001–2009	38	60	–
Holck (2013) [48]	USA (Alaska)	Alaska Native	138,312	2004–2008	252	–	42.4
Region of the Americas (USA, Lower 48 States + Hawaii)							
Levy (1965) [117]	USA (New Mexico)	Navajo	87,000	1954–1963	59	8.3	–
Kalish (1968) [118]	USA (Hawai‘i)	Kānaka Maoli (Native Hawaiian)	–	1959–1965	–	17.8	–
		Other Pacific peoples	–	1959–1965	–	6.8	–
Conrad (1974) [119]	USA (Arizona)	Tohono O’odham	12,179	1967–1971	10	–	18
Shore (1975) [120]	USA (Pacific Northwest)	American Indian	23,921	1969–1971	20	27.8	–
Sievers (1975) [121]	USA (Arizona)	American Indian	40,361	1971–1973	17	16.8	–
		Apache	–	1971–1973	–	40	–
		Akimel O’odham	–	1971–1973	–	7	–
		Other American Indian tribes	–	1971–1973	–	26	–
Miller (1979) [122]	USA (Southwest)	American Indian	–	1977	–	57.8	–
Humphrey (1982) [123]	USA (North Carolina)	Cherokee	–	1974–1976	–	31.1	–
		Lumbee	–	1974–1976	–	10.3	–
Broudy (1983) [124]	USA (Mexico and Arizona)	American Indian	162,303	1975–1977	–	–	28.5
Simpson (1983) [125]	USA (Northeastern Arizona)	Hopi	9406	1979–1980	5	27	–
Levy (1987) [126]	USA (Northern Arizona)	American Indian	7600	1981	–	23.7	–
Copeland (1989) [127]	USA (Florida)	American Indian	11,050	1982–1986	1	11	–
Sievers (1990) [128]	USA (Arizona)	Akimel O’odham	4915	1975–1984	26	53	51
Van Winkle (1993) [15]	USA (New Mexico)	Apache	–	1980–1987	179^h^	–	48.8
		Navajo	58,936	1980–1987		–	18.2
		Pueblo	–	1980–1987		–	32.2
Wissow (2001) [129]	USA (Southwest)	American Indian	12,000	1985–1996	–	30.7	24.6
Mullany (2009) [130]	USA (Arizona)	White Mountain Apache	15,500	2001–2006	41	45.5	40
Martin (2010) [131]	USA (North Carolina)	American Indian	–	2004–2007	39	8.5	–
Christensen (2013) [132]	USA (South Dakota)	American Indian	82,073	2000–2010	236	29	28

*WHO* World Health Organization, *CSIR* crude suicide incidence rate, *SSIR* standardized suicide incidence rate, *FSM* Federated States of Micronesia, *NWT* Northwest Territories, *IHSA* Indian Health Services Area

Standardized rates were adjusted with various populations; therefore they are not directly comparable. Population *n* are based on reported estimates in each article, but may not reflect denominators used to calculate incidence

^a^General terms such as Indigenous, Pacific Peoples, or First Nation were used when a specific nation or tribe was not identifiable

^b^Indigenous tribes in Brunei Darussalam included Kedayan, Belait, Tutong, Bisya, Murut, Dusun, and Iban

^c^Rate range for 10 ethnic minority groups in Yunnan Province, China: Hui, Ha ni, A chang, Pumi, Bai, Yao, Zhuang, Miao, Meng gu, and Jing po minorities

^d^Indigenous tribes in Mato Grosso do Sul, Brazil included Kadiwe’u, Guato, Ofaie ´-Xavante, Guarani-Kaiowá, Guarani-Ñandeva, and Terena

^e^Indigenous tribes included Guarani-Kaiowá, Guarani-Ñandeva, and Terena

^f^Population and rate range included 48 states

^g^Rate range for 9 Native regional corporations in Northwest Alaska: Athna, Bering Straits, Bristol Bay, Calista, Chugach, Cook Inlet, Doyon, Koniag, and Sealaska (NANA and Arctic Slope not extracted due to duplicate data with Travis, 1984 [110])

^h^Total number of deaths for Apache, Navajo, and Pueblo populations combined

### Measure of relative effect

**Fig. 2:**
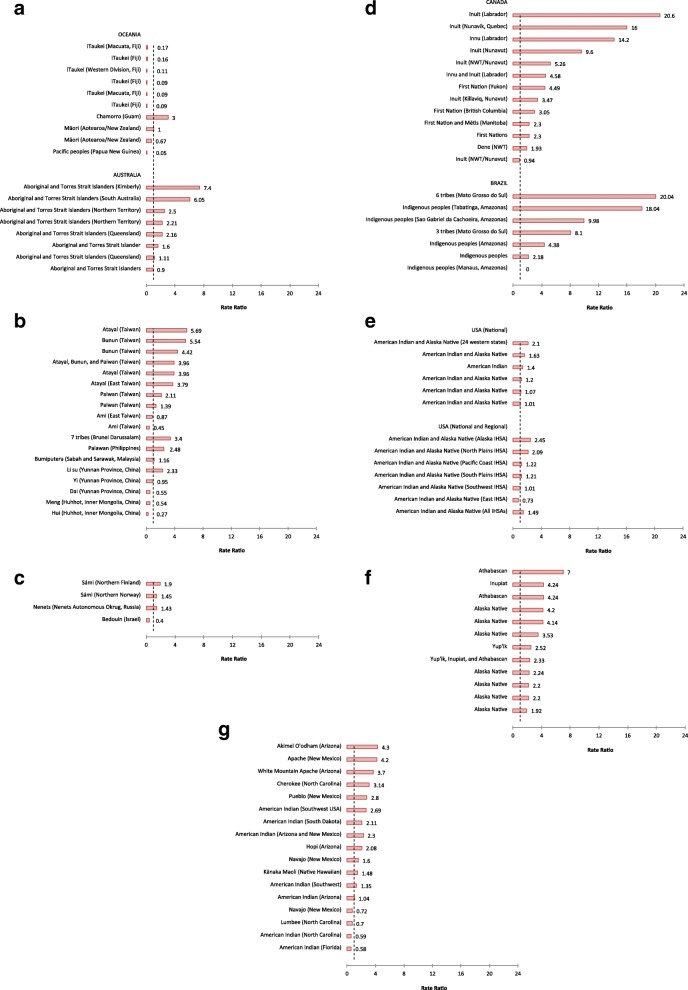
Global suicide mortality incidence rate ratios among Indigenous and comparison populations. **a** Western Pacific Region (Oceania and Australia). **b** Western Pacific Region (East Asia). **c** European Region. **d** Region of the Americas (Canada and Brazil). **e** Region of the Americas (USA, National). **f** Region of the Americas (USA, Alaska). **g** Region of the Americas (Lower 48 states and Hawaii). *NWT* Northwest Territories, *IHSA*  Indian Health Services Area. The *dotted line* indicates a rate ratio of one (RR = 1). This means that there is rate parity (no difference) between the incidence of suicide in Indigenous and comparative populations. Rate ratios to the left of the dotted line (RR < 1) indicate that rates are comparatively higher in the non-Indigenous population. Conversely, rate ratios to the right of the dotted line (RR > 1) show that the Indigenous population has a comparatively higher rate. Citations for each study are reported in Additional file 1: Supplement 3, Table S4

Incidence rate ratios were reported or calculated for 102 Indigenous populations in 69 studies. The results showed rate disparities in the majority of studies (Fig. 2), though 22 reported rate ratios below one. The rate ratios ranged from 0.04 in China [44] to more than 20 in Brazil [45] and Canada [30] (Additional file 1: Supplement 3, Table S4). Most Indigenous populations had higher suicide rates than comparison groups; disparities were widest in studies with small populations. One study reported a suicide rate of zero for an urban Indigenous population in Brazil compared the general population rate of 4.8 per 100,000 in the same city [46].

### Time trends

Suicide rates appeared to increase over time, especially in the latter half of the twentieth century, though reports were limited. Among studies with reported time series (*n* = 24), most (83%, *n* = 20) had fewer than 10 data points and covered an average of 19 years. A study in Greenland was the exception; it reported longitudinal data that showed a steady suicide rate increase among Inuit that began with the near absence of suicide in the early part of the twentieth century (2.4 per 100,000) and climbed exponentially to a rate of 110.4 per 100,000 in 2010–2011; the average number of suicides per year changed from less than one to 55 during this period [12]. Aboriginal and Torres Strait Islanders in Northern Territory, Australia experienced similar rate accelerations (6.1 per 100,000 in 1981 to 50.4 per 100,000 in 2002) [41], while incidence among Alaska Natives was relatively stable, though high, from the 1980s to the early 2000s [47, 48]. Indigenous peoples in the Micronesian islands experienced a sixfold increase in suicide rates between the 1960s and the late 1980s (from 4.3 to 25.8 per 100,000) [35], and one study reported slight rate declines for both Māori and non-Māori in New Zealand from 1996 to 2002 [5]. Annual rates tended to fluctuate in studies with small populations.

### Age differences

Age-specific rates were reported in 39 studies; various age categories were used, and rates were often only available for select strata. Youth less than 30 years old, especially those aged 15–24 years old, had the highest suicide rates of any age group in 89% of studies (*n* = 34) that reported age-specific rates. In the larger studies (> 100 total suicides) with age-specific incidence, youth suicide rates ranged from 15.9 to 108 per 100,000 population. Very few studies reported deaths or rate estimates for adults more than 60 years old.

### Gender differences

Men accounted for the majority of suicide deaths in all but four studies; only two of these four studies reported a greater number of suicide deaths among women [49, 50]. Studies with gender-specific crude and age-standardized rates (*n* = 35) ranged from zero to 75.5 per 100,000 among Indigenous women (Additional file 1: Supplement 3, Table S3). Suicide rates were higher among Indigenous men compared to Indigenous women, though rate differences were marginal among some Pacific populations [33, 51]. Suicide rates were also higher among Indigenous men than for men in comparison populations in all countries except Israel and Fiji. Outside of the relatively low rates among Indigenous men in these countries, estimates ranged from 19.5 among Sámi [13] to 248.7 per 100,000 among Inuit [30].

## Discussion

This study showed that the rate of suicide is elevated in many Indigenous populations globally, but that rate variation is common (Fig. 1). The evidence of substantial rate disparities for Indigenous peoples in Australia, Brazil, Taiwan, and circumpolar countries is notable. Equally important, we found that disparities were marginal or non-existent in some US territories and Pacific nations; we also identified 21 studies in which Indigenous populations had lower suicide rates than non-Indigenous populations. These results demonstrate that the high incidence of suicide and large rate disparities are not universal among Indigenous peoples. This confirms and extends findings from prior research that reported variation in localized estimates in the USA [52] and Canada [16].

Worldwide variation in the incidence of suicide among Indigenous peoples has complex and place-based social origins. These origins are traceable to regional differences in the impact of colonization, which is widely recognized as a major determinant of Indigenous health [17–19, 53]. Colonial governments have historically threatened the well-being of Indigenous peoples through chronic and often state-sanctioned discrimination and human rights abuses, and continue to do so in many countries [18, 20, 23]. Until 2016, several high-income countries had not ratified the United Nations Declaration on the Rights of Indigenous Peoples, and therefore legislative reforms to recognize Indigenous self-determination lagged. As a result, many Indigenous nations have yet to attain political sovereignty over lands and natural resources, education, or health care.

Globally, Indigenous peoples commonly experience social and economic marginalization and, as a consequence, some of the most disparate health outcomes [17, 18, 53]. In this context, the extent and the persistence of high suicide rates and rate disparities reveal a striking deficit in the global effort to prevent suicide and achieve social and health equity. This is further challenged by overlapping barriers to accessing health care and community supports, especially in rural areas and low- and middle-income countries. Barriers include fragmented care networks, lack of access to services due to geography, discriminatory attitudes from health care providers, and services that are not culturally safe or provided in the necessary language [18, 54, 55]. In resource-limited and conflict settings in particular, mental health services are inadequate in scope and quality, chronically under-funded, and in some places non-existent [18, 54].

Challenges in accessing mental health care are compounded by the limited relevance and generalizability of some “best practice” interventions in Indigenous contexts [56, 57]. Recent clinical trials with gatekeeper training [57], hospital-based interventions [58], and mobile self-help applications [59] reported adverse and limited effects on suicide-related outcomes for Indigenous peoples. Overall, intervention studies with Indigenous populations are rare, and community-based programs are often not evaluated or have weak study designs [60–63]. These challenges point to a need to expand efforts to generate Indigenous-specific evidence [23, 56, 60]. Indeed, many communities have developed contextualized and complex approaches to suicide prevention that respond to local priorities. There is emergent evidence that such programs increase protective factors and reduce suicide-related behavior [63–65]. However, knowledge about programs’ effectiveness, implementation, and capacity to scale up is limited, and many programs are not sustainably funded [56, 60–62].

Indigenous organizations and governments in New Zealand, Canada, and several Arctic states have moved beyond programmatic approaches and designed Indigenous-specific suicide prevention strategies [23, 55, 66]. These strategies integrate evidence-based public health and clinical interventions with Indigenous knowledge about the consequences of colonization, institutionalized violence and racism, and the value of culture. They also recognize that social conditions have an important role in shaping mental health, especially during the early years of life, and that improving these conditions can have a positive impact on population mental health and suicide-related outcomes. The path to lowering the incidence of suicide among Indigenous peoples and achieving health equity requires broader social transformation both within states and globally. This transformation must be collaborative, with Indigenous organizations and communities as leaders and rights-holders in knowledge production and decision-making [23, 29, 53, 56, 66, 67]. Public health systems can also enhance capacity for Indigenous suicide prevention with efforts to increase the visibility of community-level differences in health status and by accurately tracking changes in suicide mortality over time.

### Limitations

This study is a comprehensive synthesis of the published evidence on the global epidemiology of suicide among Indigenous peoples. Although it is the first review of this scale, our study has several important limitations. First, included studies varied their methods of identifying Indigenous populations. Self-identification is the gold standard in administrative and registry data [67]. However, this is a recent benchmark. Its uptake has varied internationally, and some countries do not identify Indigenous populations in health data at all [53, 67]. The majority of included studies relied on linkages with census or registry data, geographic proxies, or observer-determined assessments. These procedures are useful approximations, but they use varied definitions and tend to under-count Indigenous people, especially groups without legal recognition [29, 53, 67]. This can lead to ascertainment bias and underestimation of inequities [53, 67]. A second and related limitation is the under-representation of studies from low- and middle-income countries. In our review, we may have missed studies, particularly from the Global South, due to the conceptualization of Indigenous and the search terms used, which do not necessarily apply in all contexts. We attempted to limit this bias by searching databases focused on low- and middle-income countries and including non-English language papers.

The third limitation was that it was difficult to compare suicide rates between countries. Included studies were heterogeneous in population size, number of cases, aggregation, data source and outcome assessment, method of identifying Indigenous peoples, and coverage period. Many papers provided crude estimates only and did not report numerator and denominator data by age group, gender, or ethnicity. For studies with adjusted rates, different standard populations were used, and confidence intervals were rarely reported. Differences in analytic and reporting practices made it challenging to directly and reliably compare suicide rates across studies. To address this, we examined rate ratios to assess relative differences between Indigenous and non-Indigenous/general populations. This allowed us to estimate rate disparities, which were compared globally.

The fourth limitation was that studies reporting low suicide rates may be under-represented, which is a potential publication bias. It is unclear whether the lack of low incidence populations is related to the common finding of elevated rates of suicide among Indigenous peoples compared to non-Indigenous populations or, as we suspect is more likely, to the possibility that suicide rates are rarely studied when they are low. Additional low incidence reports may exist outside of peer-reviewed studies; however, these were not identified because we did not search the gray literature. The primary reason for excluding gray literature reports was the extensive volume of sources with variable quality and also the risk of over-including data from high-income nations where public reporting of mortality data is common and vital statistics infrastructure is of high quality. Nonetheless, we identified 23 papers that reported rate parity or had a rate ratio below one, but these tended to use older data. A related problem is that case studies tended to examine suicide clusters in small populations [42, 68]. The advantage of using localized data is the ability to contextualize a complex health issue. The disadvantage is that the potential to compare health status between multiple groups, across regions, and over time is reduced.

### Strengthening surveillance in Indigenous suicide prevention

Our results substantiate previous work [16, 52] to demonstrate that elevated suicide rates are not universal among Indigenous people and debunk notions that Indigeneity increases risk for suicide. Our results also point to several gaps in knowledge about the epidemiology of suicide in Indigenous populations globally. The lack of published suicide data on Indigenous populations in low- and middle-income countries is a glaring absence. Previous studies noted a scarcity of Indigenous-specific data in the Global South overall [18, 53]. Poor infrastructure for death registration is a key limitation [1]. In the context of suicide, this is especially problematic, because countries in Asia, Africa, and Latin and South America are the homelands for the majority of the world’s Indigenous populations [18] and, at a national level, account for more than three quarters of all suicide deaths [1]. Suicide data in high-income countries tends to be of better quality than that in low- and middle-income countries; however, many governments do not include Indigenous or other ethnic identifiers in administrative health data, and do not routinely link census or Indigenous registries with national health datasets such as vital statistics. In Canada for example, the federal government does not know how many Indigenous people die by suicide in a given year. Globally, there is a critical need to strengthen capacity for surveillance in Indigenous suicide prevention.

National governments can take several steps to improve suicide surveillance in Indigenous populations. Actions should include efforts to enhance suicide data quality and standardized classification by improving vital registration infrastructure, especially in low- and middle-income countries, and integrating mortality data with monitoring of suicide attempts [1]. Countries should adopt an equity-based approach to data collection that includes Indigenous identifiers derived from self-reported sources and linked to registries or census data to address gaps in identification, and align Indigenous identification procedures with recommendations from the International Group for Indigenous Health Measurement, adapted for each national context [1, 53, 56, 67, 69]. Building inclusive, Indigenous-centered models of data governance in suicide prevention will be a critical element of strengthened surveillance. To achieve this will require national statistical agencies to not only consult Indigenous communities, organizations, and leaders about priorities, but to respect Indigenous rights to determine the parameters of data ownership, custodianship, access, and use [29, 32, 67].

Future research and global suicide surveillance efforts will be further strengthened with longitudinal and up-to-date national and state-level datasets that allow disaggregation and comparisons of outcomes in small areas and subpopulations by ethnicity [1, 17, 53, 56]. Overall, these actions will help maintain robust public health surveillance systems in order to monitor health status, increase knowledge about the social determinants of suicide, target interventions, and evaluate strategies aimed at reducing the incidence of suicide among Indigenous peoples worldwide [1, 56]. Increasing the visibility of populations that bear the greatest burden from suicide can help drive efforts to achieve the WHO and Sustainable Development Goals of reducing national suicide rates by up to 30% [1, 69].

## Conclusions

Suicide among Indigenous peoples is not a universal or intractable problem. Our study showed substantial global rate variation, with striking disparities in some countries. Efforts to understand these differences and to continue to build the knowledge base for effective interventions will require sustained political and financial investments in Indigenous communities, health systems, and governments. Across sectors and countries, Indigenous peoples have called for suicide prevention strategies that are community-led, strengths-based, and trauma-informed, and that redress intersecting forms of structural discrimination, social inequity, and their downstream consequences. Global efforts to reduce suicide rates among Indigenous peoples must include actions focused on communities that experience the most profound disparities, while also seeking to promote population mental health and improve health equity.

## Additional file

Additional file 1: Supplements 1-4 (Study Protocol, Methods, Results, and PRISMA Checklist). (DOCX 595 kb)

## Abbreviations

CI: Confidence interval
CSIR: Crude suicide incidence rate
FSM: Federated States of Micronesia
IHSA: Indian Health Services Area (USA)
NWT: Northwest Territories (Canada)
SSIR: Standardized suicide incidence rate
UN: United Nations
USA: United States of America
WHO: World Health Organization
