# Supplementary material for: Global incidence of suicide among Indigenous peoples: a systematic review
Source: BMC Med. 2018 Aug 20;16:145. doi: 10.1186/s12916-018-1115-6 (PMC6100719; doi:10.1186/s12916-018-1115-6)
Supplement: Supplementary file 1 — Supplements 1-4 (Study Protocol, Methods, Results, and PRISMA Checklist). (DOCX 595 kb) [file 12916_2018_1115_MOESM1_ESM.docx]

**ADDITIONAL FILE 1: Supplements 1-4 (Study Protocol, Methods, Results, and PRISMA Checklist)**

Global incidence of suicide among Indigenous Peoples: a systematic review (Supplement)

**Authors**

Nathaniel J Pollock, Kiyuri Naicker, Alex Loro, Shree Mulay, Ian Colman

**Correspondence to:**

Nathaniel Pollock

Labrador Institute, Memorial University, Happy Valley-Goose Bay, Newfoundland and Labrador, A0P 1E0, Canada

nathaniel.pollock@med.mun.ca

**Table of Contents**

1. Supplement 1: Study Protocol
2. Supplement 2: Methods
3. Supplement 3: Results
4. Supplement 4: PRISMA Checklist

**Additional File 1: Supplement 2 (STUDY PROTOCOL)**

**Title**

The global burden of suicide among Indigenous populations: a systematic review

**Team Members**

Nathaniel J Pollock, Kiyuri Naiker, Alex Loro, Shree Mulay, and Ian Colman

**Correspondence to:**

Nathaniel Pollock

Labrador Institute, Memorial University, Happy Valley-Goose Bay, Newfoundland and Labrador, A0P 1E0, Canada

nathaniel.pollock@med.mun.ca

**Consulting Librarians**

Lindsay Alcock, Memorial University

Lindsey Sikora, University of Ottawa

**Background**

Worldwide, Indigenous peoples experience substantial health inequities compared to non-Indigenous populations.^1,2^ In high-income nations, the gap is largely due to non-communicable diseases and injuries,^1^ whereas in low- and middle-income nations, disparities are due to infant mortality and communicable diseases.^1,2^ In some high-income countries in particular, Indigenous populations experience a substantially elevated rates of suicide.^3-6^ Previous reviews have synthesized the literature for specific populations and regions,^3,4,7-13^ but have not provided a systematic and global investigation of suicide incidence amongst Indigenous populations.

**Objectives**

The objective of this study is to conduct a systematic review of the peer reviewed literature on suicide epidemiology amongst Indigenous populations globally. This study aims to report the incidence rates of suicide mortality in Indigenous populations, and compare rates to general and non-Indigenous populations to assess relative disparities. This review will report results in accordance with the PRISMA guidelines.^14^

**Questions**

This systematic review is guided by the following questions:

1. What are the patterns in suicide mortality in Indigenous populations worldwide?
2. What is the incidence rate ratio of suicide among Indigenous populations compared to general or non-Indigenous populations?

**Search Strategy**

We will combine search terms related to three concept areas: population (Indigenous), outcome (suicide), and study design (observational). Key and MeSH Term selection to identify global Indigenous populations is based on previous reviews in Indigenous health.^11,15-21^ We will include terms related to geographic regions (ex. Greenland) because some studies use regions or countries with a high proportion of Indigenous people as a proxy for Indigenous status, and mortality data in many countries does not include individual-level Indigenous identifiers.

**Search Terms**

We will use the following search terms adapted for each database:

Table 1: Search Terms

| **Concept 1 (Population)** | **Concept 2 (outcome)** | **Concept 3 (Study design)** |
| --- | --- | --- |
| "first nation" | suicid* | "ecological study" |
| "first nations" |  | cohort |
| "pacific islander" | suicide [mesh] | "case control" |
| "pacific islanders" |  | "observational" |
| "torres strait islander" |  | "population-based" |
| "torres strait islanders" |  | epidemiolog* |
| aborigin* |  | incidence |
| africa* |  | mortality |
| alaska* |  |  |
| aleut* |  | epidemiologic studies [mesh] |
| amerind* |  | incidence [mesh] |
| arctic |  | mortality [mesh] |
| aymara |  |  |
| bushmen |  |  |
| chukchi |  |  |
| chukotka* |  |  |
| circumpolar |  |  |
| eskimo* |  |  |
| greenland* |  |  |
| hmong |  |  |
| indian* |  |  |
| indigen* |  |  |
| inuit* |  |  |
| inupiaq |  |  |
| inupiat |  |  |
| khanty |  |  |
| maori* |  |  |
| mapuche |  |  |
| metis |  |  |
| native* |  |  |
| navaho* |  |  |
| navajo* |  |  |
| nenets |  |  |
| quechua |  |  |
| saami |  |  |
| sami |  |  |
| samoan* |  |  |
| siberia |  |  |
| skolt |  |  |
| tribal |  |  |
| tribe* |  |  |
| xingu* |  |  |
| yup'ik |  |  |
| yupik |  |  |
| zuni |  |  |
|  |  |  |
| "African continental ancestry group" [Mesh] | |  |
| "American Native continental ancestry group" [Mesh] | |  |
| "Asian continental ancestry group" [Mesh] | |  |
| "Health Services, Indigenous"[Mesh] | |  |
| "Oceanic ancestry group"[Mesh] | |  |
| "arctic regions" [Mesh] |  |  |
| "ethnic groups" [mesh] |  |  |

**Search Databases**

We will search the following databases with a search string customized for each based on the listed terms:

1. PubMed
2. MEDLINE
3. Embase
4. CINAHL
5. PsycINFO
6. Lilacs
7. SciELO

We will perform text word and Medical Subject Heading searches in each database as applicable. We will also hand-search the reference lists of included studies and previous reviews for additional eligible articles. Finally, we will conduct secondary searches in World Health Organization regional literature databases and select publications and databases focused on Indigenous health.

**Types of studies to be included**

This systematic review will include primary, observational studies of population-based data. Case reports, editorials and commentaries, reviews, qualitative designs, program evaluations, intervention or experimental studies, articles that have not been peer-reviewed or are not primary research, iterations, and those with duplicate data will also be excluded. We will not examine studies in the grey literature.

**Inclusion Criteria**

1. Design: Observational, population-based study that includes a cohort element in the design;
2. Population: Indigenous population that is identifiable at individual level or by geographic proxy;
3. Outcome: crude or standardized suicide mortality incidence rates;
4. Includes at minimum the population aged 15-65 years old.

**Exclusion Criteria**

1. Design: Case report/series, qualitative, review, editorial, intervention/experimental/program evaluation;
2. Not peer reviewed;
3. Not primary research (e.g. studies that re-reported rates calculated by another source or did not conduct original data analysis);
4. Indigenous people are not identifiable as a group for comparison (e.g. studies with area-based estimates where the Indigenous peoples accounted for less than 80% of the total population);
5. Study reporting on a specific strata only (e.g. specific method, age group, or sex);
6. Clinical subpopulation (e.g. prisoners, substance users);
7. Rates and count data in figures only.

We will not exclude papers on the basis of language, though we may miss papers written and indexed in non-European languages.

**Condition or domain being studied**

Suicide mortality

**Population**

Indigenous

Although there is no global consensus on the definition of Indigenous for the purpose of this review we will use the United Nations’ ‘working definition.^1,2^ *Indigenous* is conceptualized as involving:

- Self-identification;
- Recognition by community;
- Connection to a specific territory;
- Distinct political, economic and social systems;
- Distinct language, culture, and beliefs;
- Form non-dominant groups of society;
- Maintain ancestral connections with environment and systems as a distinct people;
- Historical continuity with pre-colonial societies.

**Intervention(s)**

Not applicable.

**Comparator(s)/Control**

This review will consider non-Indigenous or general populations for the comparison groups, though a comparison population is not a requirement for inclusion.

**Outcome(s)**

*Primary*

Crude or age-standardized incidence rate.

*Secondary*

Measure of relative effect (incidence rate ratio).

If crude rates are not reported, but numerator (suicide deaths) and denominator (population) are provided, we will calculate rates. Similarly, for studies that do not report an incidence rate ratio but provide a comparator rate (or numerator and denominator data), we will calculate the rate ratio.

**Data extraction (selection and coding)**

Two authors will independently validate the search strategy. One author will remove duplicates, and two authors will then screen the titles and abstracts of all articles and remove those that are unambiguously ineligible. Two teams (with two authors in each) will review the full text of all screened-in articles to assess for eligibility against the inclusion and exclusion criteria. Any disagreements between authors on article eligibility will be resolved by discussion with the third author to reach consensus. Two authors will extract information from each eligible study using a standardized data extraction form developed for this study. The following details will be obtained from each paper: citation, country, design, sample characteristics (Indigenous group, age groups included, population count by gender and overall), comparison population, data source, study period, number of deaths, crude and standardized incidence rates, rate ratio, and confidence intervals.

**Protocol for Title/Abstract Screening and Full Text Review**

The title/abstract screening process includes 3 yes/no questions based on the study inclusion criteria. If all questions are answered ‘yes’ or ‘maybe’ for a given article, then it will be included in the full text review; ‘no’ answers result in the article being screened out. If the questions cannot be answered based on the abstract, then the article will be included in the full text review. The following questions will be used to assess eligibility:

1. Does the article study an identifiable Indigenous population or a region with a predominantly Indigenous population (example: Greenland)?
2. Does the study use an observational design with population-based data as defined by the inclusion criteria?
3. Does the article report a suicide incidence rate for the Indigenous population or report original count and population data?

**Risk of bias (quality) assessment**

Two authors will independently assess each included full text article using a modified version of the Ottawa-Newcastle Quality Assessment Scale for Cohort Studies.^22^ This tool was selected because of its utility is assessing the quality of observational research.

**Data Synthesis**

We expect heterogeneity in the data from included studies in terms of case and population data sources used, study period, age and sex stratification, population size, and comparators. Therefore, we anticipate that a meta-analytic approach to pooling the data may not be possible. We will conduct a narrative review of studies that report incidence rate estimates and rate ratios. This will include descriptions of data sources, populations, methods, and findings. We will synthesize results in figures and tables, and in a narrative report.

**Dissemination Plan**

We will submit the final manuscript for publication in a peer-reviewed journal, and present the results at a conference. The manuscript will also be included in the doctoral thesis for one of the authors (NP).

**Funding sources**

None

**Additional File 1: Supplement 2 (METHODS)**

**Title**

The global burden of suicide among Indigenous populations: a systematic review

**Authors**

Nathaniel J Pollock, Kiyuri Naiker, Alex Loro, Shree Mulay, and Ian Colman

**Correspondence to:**

Nathaniel Pollock

Labrador Institute, Memorial University, Happy Valley-Goose Bay, Newfoundland and Labrador, A0P 1E0, Canada

nathaniel.pollock@med.mun.ca

**SEARCH STRATEGY**

We combined search terms related to three concept areas: (1) population (Indigenous); (2) outcome (suicide); and (3) study design (observational). Term selection to identify global Indigenous populations was based on previous reviews in Indigenous health,^11,15-21^ and combined key and MeSH terms adapted for each database. We used a combination of general terms that are common in the literature such as “indigenous,” “native,” and “aboriginal”, geographic terms such as “arctic” and “circumpolar”, nation-, tribe-, or group-specific terms such as Mapuche, Khanty, and Sámi. We also included terms that have been used historically and presently in some countries, but are viewed as derogatory in other contexts (ex. “Eskimo”). We included terms related to geographic regions because some studies use regions with a high proportion of Indigenous peoples as a proxy for Indigenous status because mortality data in many countries does not include individual-level ethnic identifiers. We used only English language terms in the primary search, however, the truncated root of our primary search terms, “indigen*” “suicid*” are the same in English, Spanish, Portuguese, and French.

To create the search strategy, we used a stepped procedure similar to a previous systematic review.^18^ We conducted a pilot search with a preliminary list of Indigenous terms. We then removed a single term, re-ran the search, and compared the results. If the removal of a given term did not change the number of results; the removed term was deemed redundant, and removed from the final search string.

**Search Terms in PubMed**

Concept #1 (Outcome: Suicide)

(suicid*[Text Word]) OR "Suicide"[Mesh]

AND

Concept #2 (Population: Indigenous Peoples)

("first nation"[Text Word]) OR "first nations"[Text Word]) OR "pacific islander"[Text Word]) OR "pacific islanders"[Text Word]) OR "torres strait islander"[Text Word]) OR "torres strait islanders"[Text Word]) OR aborigin*[Text Word]) OR africa*[Text Word]) OR alaska*[Text Word]) OR aleut*[Text Word]) OR amerind*[Text Word]) OR arctic[Text Word]) OR aymara[Text Word]) OR bushmen[Text Word]) OR chukchi[Text Word]) OR chukotka*[Text Word]) OR circumpolar[Text Word]) OR eskimo*[Text Word]) OR greenland*[Text Word]) OR hmong[Text Word]) OR indian*[Text Word]) OR indigen*[Text Word]) OR inuit*[Text Word]) OR inupiaq[Text Word]) OR inupiat[Text Word]) OR khanty[Text Word]) OR maori*[Text Word]) OR mapuche[Text Word]) OR metis[Text Word]) OR native*[Text Word]) OR navaho*[Text Word]) OR navajo*[Text Word]) OR nenets[Text Word]) OR quechua[Text Word]) OR saami[Text Word]) OR sami[Text Word]) OR samoan*[Text Word]) OR siberia*[Text Word]) OR skolt[Text Word]) OR tribal[Text Word]) OR tribe*[Text Word]) OR xingu*[Text Word]) OR yup’ik[Text Word]) OR yupik[Text Word]) OR zuni[Text Word]) OR "African continental ancestry group"[Mesh]) OR "African continental ancestry group"[Mesh]) OR "Asian continental ancestry group"[Mesh]) OR "Health Services, Indigenous"[Mesh]) OR "Oceanic ancestry group"[Mesh]) OR "arctic regions"[Mesh]) OR "ethnic groups"[mesh]

AND

Concept #3 (Study Design: Observational)

("ecological study"[Text Word]) OR "case control"[Text Word]) OR cohort[Text Word]) OR observational[Text Word]) OR "population based"[Text Word]) OR epidemiolog*[Text Word]) OR incidence[Text Word]) OR mortality[Text Word]) OR "epidemiologic studies" [Mesh]) OR "incidence" [Mesh]) OR "mortality" [Mesh]

We conducted an secondary search to compare results based on the included search terms with an expanded and comprehensive list of terms identified in previous reviews.^11,15-19,21^ This secondary search included terms for specific tribal groups and nations such as Innu, Batwa, Maasai, and Cherokee. No additional eligible studies were identified; this outcome validated the original search strategy.

Throughout the paper, to we refer specific nations, tribal groups, or peoples (ex. Navajo). When this is not possible, we use country- or region-specific terms (ex. Aboriginal and Torres Strait Islander), or the general term, Indigenous peoples.

**Databases**

We conducted computerized searches of the following electronic literature databases from inception until June 1, 2017: PubMed, MEDLINE, Embase, CINAHL, PsycINFO, Lilacs, and SciELO. In effort to identify additional studies, especially those with data from low-and-middle income countries, we hand searched the reference lists of previous reviews on suicide in Indigenous populations, and select reviews of suicide epidemiology in Africa and South Asia.^3,4,7-13,20,23-50^ We also conducted searches with the term “suicide” in all fields in supplementary databases and peer reviewed journals focused on Indigenous peoples or regional populations. The following supplementary sources were examined:

*Supplementary Databases:*

1. Bibliography of Native Americans (EBSCO)
2. Arctic and Antarctic Regions (EBSCO)
3. Indigenous Studies Portal (University of Saskatchewan)
4. Circumpolar Health Bibliographic Database (University of Alberta)
5. International Journal of Circumpolar Health Special Issue (2015, Volume 73, Issue 1): Suicide and Resilience in Circumpolar Populations
6. WHO Journal Database: Western Pacific Region Index Medicus
7. WHO Journal Database: African Index Medicus
8. WHO Journal Database: Index Medicus for South-East Asian Region

*Peer-Reviewed Journals:*

1. Journal of Aboriginal Health/International Journal of Indigenous Health
2. Pimatisiwin: A Journal of Aboriginal and Indigenous Community Health
3. Aboriginal Policy Studies
4. White Cloud: Journal of American Indian and Alaska Native Mental Health

**Types of studies to be included**

This systematic review included primary, observational studies of population-based data. We excluded case reports, editorials and commentaries, reviews, qualitative designs, program evaluations, intervention or experimental studies, articles that were not peer-reviewed or not primary research, iterations, and those with duplicate data. We did not examine studies reported in books, theses/dissertations, government reports, or elsewhere in the grey literature.

***Inclusion Criteria***

1. Design: Observational, population-based study that included a cohort element;
2. Population: Indigenous population that is identifiable at individual level or by geographic proxy;
3. Outcome: crude or standardized suicide mortality incidence rates;
4. Included at minimum the population aged 15-65 years old.

***Exclusion Criteria***

1. Design: Case report/series, qualitative, review, editorial, intervention/experimental/program evaluation;
2. Not peer reviewed;
3. Not a primary research (e.g. studies that re-reported rates calculated by another source or did not conduct original data analysis);
4. Study focused on specific subgroup (specific method, age group, or sex);
5. Clinical subpopulation (i.e. prisoners, substance users);
6. Indigenous people are not identifiable as a group for comparison (e.g. studies with area-based estimates where the Indigenous peoples accounted for less than 80% of the total population)

We did not exclude papers on the basis of language, though we may have missed papers that were written and indexed in non-European languages.

***Protocol for Abstract/Title Screening and Full Text Review***

The abstract/title screening process included three yes/no questions based on the study inclusion criteria. If all questions were answered ‘yes’ or ‘maybe’ for a given article, then it was included in the full text review; ‘no’ answers resulted in the article being screened out. If the questions could not be answered based on the abstract, then the article was included in the full text review. The following questions were used:

1. Does the article study an identifiable Indigenous population or a region (or territory/state) with a predominantly Indigenous population (ex. Vanuatu, Greenland, etc.)?
2. Does the article use an population-based, observational design with a cohort element?
3. Does the article report a suicide mortality incidence rate for the Indigenous population or provide numerator and denominator data?

During the title and abstract screening stage, we excluded papers based on the follow-criteria:

1. Duplicates;
2. Not peer reviewed;
3. Did not examine suicide mortality;
4. Did not examine a specific Indigenous population or geographic proxy >80%;
5. Did not include a cohort element in the study design;
6. Examined only suicide deaths by a specific method (i.e poisoning, firearm);
7. Was not a population-based study (i.e. focused on a specific subpopulation only such as prisoners, substance users, men, or children).

During the full text review stage, we further excluded papers based on the following related criteria:

1. Did not include, at minimum, age groups between 15-65 years;
2. Did not include an identifiable Indigenous population or geographic proxy at the individual or ecological level;
3. Did not include crude, age-specific or adjusted suicide mortality rates or sufficient data (count and population) in numerator and denominator to estimate rates for an Indigenous population;
4. Reported rates in figures only;
5. Did not conduct the primary data analysis (i.e. systematic review or reproduced rates from a government report) and/or was not a primary study; and
6. Reported incidence rates from same dataset and with a period of overlap as another paper, but was less comprehensive (i.e. examined a shorter period).

**Data extraction and Analysis**

Two authors extracted information from each eligible study using a standardized data extraction form developed for this study. In the absence of a reported incidence rate (IR), we calculated crude incidence rates (number of deaths/population) if numerator (suicide deaths, n=) and denominator (Indigenous population count, n=) data was provided in accordance with inclusion criteria. Similarly, when the measure of relative effect was not reported, we calculated an incidence rate ratio (IRR) if a rate was reported or derived for an Indigenous population and a comparison population. The rate ratio was calculated as IRR=IR_Indigenous_/IR_Comparison_ and reported in figures and tables.

**QUALITY ASSESSMENT**

We conducted quality assessments of included studies with a modified version of the Newcastle-Ottawa Scale (NOS) Quality Assessment Scale for Cohort Studies.^22^ We selected this tool because of the utility for assessing the quality of observational research, and adapted the scale to reflect the specific needs of our study. We modified the scale by removing two items that were not applicable to our study (*Selection* item #2: selection of non exposed cohort; *Selection* item #4: demonstration that outcome of interest was not present at start of study). Three items from the NOS corresponded with study eligibility criteria: *Selection* item #1: representativeness of the exposed cohort; *Outcome* item #2: was the follow-up long enough to occur; and *Outcome* item #3: adequacy of follow-up of cohorts. All included studies scored a point for these items by virtue of eligibility for inclusion in the review. Therefore, we assessed quality based on a the following items from each domain, adapted for the study:

SELECTION (1 point max.)

1. Ascertainment of exposure (Data source and method of determining Indigenous identity)
2. secure record (e.g., administrative source or registry), self report (e.g. linked to census), or community informant (e.g. interview with family) **= 1 point**
3. geographic proxy
4. no description

COMPARABILITY (1 point max.)

1. Comparability of cohorts on the basis of design or analysis (
2. study controlled for age **= 1 point**
3. study reported crude rates only
4. no description

OUTCOME (2 point max.)

1. Assessment of outcome (Data source)
2. independent blind assessment: Physician certification or medico-legal records (death certificate in vital statistics, medical examiners report, other governmental registry, or coroner/police records) **= 1 point**
3. verbal report, verbal autopsy by non-physician, or other field work
4. no description
5. Adequacy of Ascertainment of suicide data
6. Near complete capture of all cases of suicide in population (Linked and cross-referenced multiple data sources) **= 1 point**
7. Cases identified with a single, routinely collected data source (some misclassification or under-reporting expected) **= 1 point**
8. Specific threat to capture of suicide cases is reported

We accounted for limitations related to under-counting of Indigenous peoples in the quality assessments. Studies that used data with Indigenous self-identification were rated the highest quality, whereas studies with geographic proxy or observer-determined Indigenous status, were rated the lowest. To assess the comparability of results within each paper, we selected age as the primary factor and assigned a score of 1 in this domain for studies that adjusted for this factor in their rate estimation.

Our modified scale includes the assessment of three domains (Selection, Comparability, and Outcome), with a maximum score of one point each for the first two domains, and two points for Outcome. On the modified four point scale, we considered papers with a score of 1 or less to be poor quality, two to three of moderate quality, and four of high quality. Two authors independently assessed each study with the modified NOS. To resolve disagreements, we reached consensus through discussion or by consultation with a third author.

**Additional File 1: Supplement 3 (RESULTS)**

**Supplemental Tables 1-4**

1. **Supplemental Table 1: Characteristics of included studies**

| **First Author (Year)** | **Country (Region)** | **Indigenous Peoples, Nation, or Tribe*** | **Indigenous Population estimate** | **Data source for Indigenous identity of decedents** | **Comparison Population** | **Age limits†** | **Mortality data source‡** | **Type of rate (Standard population)** |
| --- | --- | --- | --- | --- | --- | --- | --- | --- |
| **International, n=4** | | | | | | | | |
| Stevenson (1998)^51^ | Australia and USA | American Indians, Aboriginal and Torres Strait Islander (excluding Queensland) | n/r | Death certificate | Non–Indigenous (USA and Australia) | All ages | Registry | Age adjusted (WHO Standard Population) |
| Bramley (2004)^52^ | International (Aotearoa/New Zealand, Australia, Canada, and USA) | Maori, Aboriginal and Torres Strait Islander, First Nation (“on reserve”), and American Indian and Alaska Native | n/r | Varies by country (Linkage with census, vital statistics, and administrative registry) | Non–Indigenous | All ages | Registry | Age adjusted (World Standard Population) |
| Hezel (1989)^53^ | International (Federated States of Micronesia [Yap, Chuuk, Pohnpei, Kosrae], Palau, and Marshall Islands) | Yapese, Chuukese, Pohnpeian, Kosraen, Palauan, Marshallese | 142,298^¶^ | Geographic proxy | n/r | All ages | Linked | Crude |
| Booth (1999)^54^ | International (Papua New Guinea, Fiji, Vanuatu, FSM [Pohnpei, Kosrae, Yap, and Chuuk], Marshall Islands, Palau, American Samoa, French Polynesia, and Western Samoa) | Pacific peoples (iTuakei, ni–Vanuatu, Yapese, Chuukese, Pohnpeian, Kosraen, Marshallese, Palauan,Samoan, Polynesian) | Range: 16,500–4,216,100^§^ | Geographic proxy for all states, except iTuakei (Fijians) in Fiji | Guam | ≥5 years | Police records, health records, case studies, and coroner | Age adjusted (WHO Standard Population) |
| **European Region, n=8** | | | | | | | | |
| Soininen (2008)^55^ | Finland (Northern) | Sámi | 2,091 | Linked to self–reported identity in census | Non–Sámi | All ages | Registry | Crude |
| Bjerregaard (2015)^56^ | Greenland | Kalaallit (Inuit) | 57,000 | Geographic proxy | n/r | ≥10 years | Linked | Crude |
| Thorslund (1989)^57^ | Greenland | Kalaallit (Inuit) | n/r | Place of birth in vital registration is a proxy identifier | n/r | All ages | Linked | Crude |
| Bjorksten (2005)^58^ | Greenland (West region) | Kalaallit (Inuit) | 44,217 | Place of birth in vital registration is a proxy identifier | n/r | All ages | Registry | Crude |
| Klomek (2016)^59^ | Israel | Bedouin | n/r | Death Certificate | Arab population (Israel) | ≥15 years | Registry | Age adjusted (2009 Israel population) |
| Silviken (2009)^60^ | Norway (Northern) | Sámi | 19,801^\|\|^ | Census linked | General population (Norway) | All ages | Registry | Crude |
| Sumarokov (2014)^61^ | Russia (Nenets Autonomous Okrug) | Nenets | 7,504 | Passport and medical records | Non–Indigenous (NAO) | All ages | Linked | Age adjusted (European Standard Population) |
| Hassler (2005)^62^ | Sweden | Sámi | 41,721 | Linked administrative registry | n/r | All ages | Registry | Crude |
| **Western Pacific Region (Oceania), n=22** | | | | | | | | |
| Pridmore (2009)^63^ | Australia (Northern Territory) | Aboriginal and Torres Strait Islander | n/r | Coroner/Medical Examiner records | Non–Indigenous (Northern Territory) | All ages | Medico–legal investigation records or database | Age adjusted (n/r) |
| Measey (2006)^64^ | Australia (Northern Territory) | Aboriginal and Torres Strait Islander | n/r | Vital registration | Non–Indigenous (Northern Territory) | ≥10 years | Linked | Age adjusted (1991 Australian population) |
| Clayer (1991)^65^ | Australia (South Australia) | Aboriginal and Torres Strait Islander | 13,298 | Probabilistic identification based on name, place of residence, and local consultation | Non–Indigenous South Australia) | ≥10 years | Medico–legal investigation records or database | Crude |
| Campbell (2016)^66^ | Australia (Kimberley) | Aboriginal and Torres Strait Islander | 11,050 | Medical records | Non–Indigenous | All ages | Linked | Age and sex adjusted (n/r) |
| De Leo (2011)^67^ | Australia (Queensland) | Aboriginal and Torres Strait Islander | n/r | Psychological autopsy interview by police with family | Non–Indigenous (Australia) | ≥5 years | Linked | Age adjusted (WHO World Standard Population) |
| Cantor (1997)^68^ | Australia (Queensland) | Aboriginal and Torres Strait Islander | n/r | n/r | General population (Queensland) | 15–64 years | Linked | Age adjusted (n/r) |
| Langley (2000)^69^ | Aotearoa/New Zealand | Māori | n/r | “Sole Māori” code in Vital Statistics registry | n/r | All ages | Registry | Crude |
| Langley (1990)^70^ | Aotearoa/New Zealand | Māori | n/r | n/r | Non–Māori | ≥14 years | Registry | Age adjusted (n/r) |
| Beautrais (2006)^71^ | Aotearoa/New Zealand | Māori | 586,000 | Census linked and Interview with family or community informant | Non–Māori | All ages | Registry | Age adjusted (n/r) |
| Rubinstein (1983)^72^ | Federated States of Micronesia | Micronesian | 115,000 | Geographic proxy | n/r | ≥15 years | Linked | Crude |
| Hezel (1984)^73^ | Federated States of Micronesia (Chuuk) | Chuukese | 37,488** | Geographic proxy | n/r | ≥10 years | Linked | Crude |
| Pridmore (1995)^74^ | Fiji | iTaukei | n/r | Medical records | Indian (Fiji) | All ages | Hospital | Crude |
| Price (1975)^75^ | Fiji | iTaukei | n/r | n/r | Indian (Fiji) | All ages | Linked | Crude |
| Haynes (1984)^76^ | Fiji (Macuata) | iTaukei | 8,111 | n/r | Indian (Fiji) | ≥15 years | Police | Crude |
| Ree (1971)^77^ | Fiji (Macuata) | iTaukei | 9,950 | n/r | Indian (Fiji) | All ages | Police | Crude |
| Pridmore (1994)^78^ | Fiji (Western Division) | iTaukei | n/r | Medical records | Indian (Fiji) | All ages | Hospital | Crude |
| Booth (2010)^79^ | Guam | Chamorro | n/r | n/r | Filipino (Guam) | All ages | Registry | Crude |
| Parker (1966)^80^ | Papua New Guinea | Pacific peoples | n/r | n/r | European (Papua New Guinea) | All ages | Linked | Crude |
| Smith (1981)^81^ | Papua New Guinea (Southern Highlands) | Huli people | 50,000 | n/r | n/r | All ages | Linked | Crude |
| Pridmore (1997)^82^ | Solomon Islands (Honiara area) | Melanesian | 75,000 | Geographic proxy | n/r | All ages | Hospital | Crude |
| Vivili (1999)^83^ | Tonga | Tongan | 98,200† | Geographic proxy | n/r | All ages | Police | Crude |
| De Leo (2013)^84^ | Vanuatu | ni–Vanuatu | 245,619 | Geographic proxy | n/r | All ages | Registry | Crude |
| **Western Pacific (East Asia), n=9** | | | | | | | | |
| Telisinghe (2014)^85^ | Brunei Darussalam | Indigenous tribes including Kedayan, Belait, Tutong, Bisya, Murut, Dusun and Iban | 14,000 | n/r | Malay–Brunei | ≥10 years | Linked | Crude |
| Wang (1997)^86^ | China (Hohhot, Inner Mongolia) | Mengs, Huis | 27,000 (Mengs), 21,600 (Huis) | n/r | Han Chinese | All ages | Linked | Crude |
| Lu (2013)^87^ | China (Yunnan Province) | Dai, Yi, Lu si, Hui, Ha ni, A chang, Pumi, Bai, Yao, Zhuang, Miao, Meng gu, and Jing po ethnic minority groups | 2,977,346 | n/r | Han Chinese | All ages | Linked | Crude |
| Ali (2014)^88^ | Malaysia (Sabah and Sarawak) | Bumiputera of Sabah and Sarawak | 2,981,300 | n/r | Malay | ≥5 years | Linked | Crude |
| Jollant (2014)^89^ | Philippines | Palawan | 1,192 | Interview with family or community informant | Matched Palawan villages | All ages | Informants | Crude |
| Hsieh (1994)^90^ | Taiwan | Atayal, Bunun, Paiwan | 200,000 | Geographic proxy | General population (Taiwan) | All ages | Registry | Crude |
| Cheng (1992)^91^ | Taiwan | Atayal, Ami, Bunun, Paiwan | n/r | Interview with family or community informant | General population (Taiwan) | All ages | Registry | Crude |
| Wen (2004)^92^ | Taiwan (“Aboriginal townships”) | Indigenous peoples | 200,537 | Geographic proxy | General population (Taiwan) | All ages | Registry | Crude |
| Liu (2011)^93^ | Taiwan (East) | Ami, Atayal | n/r | n/r | Han Chinese (Taiwan) | ≥15 years | Linked | Crude |
| **Region of the Americas (Brazil and Canada), n=20** | | | | | | | | |
| Machado (2015)^94^ | Brazil | Indigenous peoples | n/r | Linked to self–reported identity in census | White population (Brazil) | ≥10 years | Registry | Crude |
| Orellana (2016)^95^ | Brazil (Mato Grosso do Sul) | Guarani–Kaiowá, Guarani–Ñandeva, and Terena | 75,000 | Linked to self–reported identity in census | Non–indigenous | ≥9 years | Registry | Age adjusted (WHO Standard Population) |
| Coloma (2006)^96^ | Brazil (Mato Grosso do Sul) | Indigenous tribes: Kadiwe'u, Guato, Ofaie ́–Xavante, Guarani–Kaiowá, Guarani–Ñandeva, and Terena | 53,325 | Census linked | General population (Brazil) | All ages | Linked | Crude |
| Souza (2013)^97^ | Brazil (Amazonas) | Indigenous peoples | 184,764^‡‡^ | Self–reported in census | Non–Indigenous (Brazil) | All ages | Registry | Age adjusted (WHO World Standard Population) |
| Mao (1992)^98^ | Canada | First Nation^¶¶^ | n/r | Linked to administrative registry | General population (Canada) | 0–64 years | Registry | Age adjusted (1981 Canadian population) |
| Isaacs (1998)^99^ | Canada (NWT) | Inuit & Dene | n/r | n/r | Non–Aboriginal and Western NWT | All ages | Medico–legal investigation records or database | Crude |
| Wotton (1985)^100^ | Canada (Labrador) | Innu and Inuit | 2,500 | n/r | General population (Canada) | All ages | Linked | Age adjusted (n/r) |
| Hislop (1987)^101^ | Canada (British Columbia) | First Nation^¶¶^ | 55,000 | Linked to administrative registry | Non–Indigenous (British Columbia) | All ages | Registry | Age adjusted (1971 Canadian Census) |
| Macaulay (2004)^102^ | Canada (Kivalliq, Nunavut) | Inuit | 7,131 | Geographic proxy | General population (Canada) | All ages | Linked | Age adjusted (1991 Canadian Standard Population) |
| Ross (1986)^103^ | Canada (Norway House Cree Nation, Manitoba) | Cree^¶¶^ | 2,822 | Linked to administrative registry | Non–Indigenous population living on reserve | All ages | Hospital | Crude |
| Chandler (1998)^104^ | Canada (British Columbia) | Native | n/r | Coroner/Medical Examiner records | General population (British Columbia) | All ages | Medico–legal investigation records or database | Crude |
| Young (1983)^105^ | Canada (Sioux Lookout Zone, Northwest Ontario) | Cree–Ojibway | 10,000 | n/r | n/r | All ages | Linked | Crude |
| Pollock (2016)^106^ | Canada (Labrador) | Innu and Inuit | Innu (1,815), Inuit (2,415) | Geographic proxy | General population (Newfoundland) | ≥10 years | Registry | Age adjusted (1991 Canadian population) |
| Fox (1984)^107^ | Canada (Wikwemikong Unceded Indian Reserve, Ontario) | Anishnaabe | 3,000 | n/r | n/r | All ages | Linked | Crude |
| Garro (1988)^108^ | Canada (Manitoba) | First Nation^¶¶^ | 43,000 | Probabilistic identification based on name, place of residence, and local consultation | n/r | All ages | Linked | Crude |
| Spaulding (1985)^109^ | Canada (Northwest Ontario) | Ojibway | 3,005 | n/r | n/r | All ages | Linked | Crude |
| Mao (1986)^110^ | Canada (7 provinces)**^§§^** | First Nations (“On Reserve”) | 168,529 | Linked to administrative registry | General population (Canada) | 1–69 years | Registry | Age adjusted (1971 Canadian Standard Population) |
| Malchy (1997)^111^ | Canada (Manitoba) | First Nations and Métis | n/r | Coroner/Medical Examiner records | Non–Indigenous (Manitoba) | ≥10 years | Medico–legal investigation records or database | Age adjusted (1991 Canadian Census population of Manitoba) |
| Penney (2009)^112^ | Canada (Nunavik, Quebec and Nunavut) | Inuit | 20,489 (Nunavut), 7,628 (Nunavik) | Geographic proxy | n/r | All ages | Registry | Age adjusted (2001 Inuit population) |
| Butler (1965)^113^ | Canada (Yukon, NWT, Nunavut) | Inuit and First Nation | 15,440^\|\|\|\|^ | n/r | Non–Indigenous (Yukon, NWT, Nunavut) | All ages | Linked | Crude |
| **Region of the Americas (Alaska, USA), n=12** | | | | | | | | |
| Marshall (1998)^114^ | USA (Alaska) | Yup'ik, Inupiat, and Athabascan | 25,000 | n/r | General population (Alaska) | All ages | Medico–legal investigation records or database | Crude |
| Kraus (1979)^115^ | USA (Alaska) | Alaska Native | 56,477 | n/r | General population (USA) | ≥15 years | Registry | Crude |
| Kettl (1991)^116^ | USA (Alaska) | Alaska Native | n/r | n/r | General population (USA) | All ages | Registry | Crude |
| Holck (2013)^117^ | USA (Alaska) | Alaska Native | 138,312 | n/r | White population (USA) | All ages | Registry | Age adjusted (2000 US Standard Population) |
| Hlady (1988)^118^ | USA (Alaska) | Alaska Native | n/r | n/r | White population (Alaska, USA) | All ages | Linked | Age Adjusted (n/r) |
| Forbes (1988)^119^ | USA (Alaska) | Alaska Native | n/r | n/r | White population (Alaska, USA) | ≥15 years | Registry | Age adjusted (1988 US Census) |
| Day (2009)^120^ | USA (Alaska) | Alaska Native | 97,012 | n/r | White population (Alaska, USA) | All ages | Registry | Age adjusted (2000 US Standard Population) |
| Day (2003)^121^ | USA (Alaska) | Alaska Native | 91,300 | Death certificate | White Population (USA) | All ages | Registry | Age adjusted (1940 Standard million) |
| Andon (1997)^122^ | USA (Alaska) | Athabascan | 6,041 | Linked to administrative registry | American Indians | 5–65 years | Linked | Crude |
| Travis (1983)^123^ | USA (Northwestern Alaska) | Alaska Native | n/r | n/r | n/r | All ages | Linked | Crude |
| Travis (1984)^124^ | USA (NANA and Arctic Slope, Alaska) | Inupiat | 7,345 | n/r | n/r | All ages | Linked | Crude |
| Wexler (2012)^125^ | USA (Northwestern Alaska) | Alaska Native | 7,965 | n/r | n/r | All ages | Linked | Age and sex adjusted (2000 US Census, Northwest Alaska) |
| **Region of the Americas (USA), n=24** | | | | | | | | |
| Lester (1994)^126^ | USA | American Indian | n/r | n/r | White population (USA) | ≥15 years | Registry | Crude |
| Howard (2014)^127^ | USA | American Indian and Alaska Native (non–Hispanic) | >2,000,000 | n/r | Non–Hispanic White population (USA) | All ages | Registry | Age adjusted (2000 US Standard Population) |
| Young (1993)^128^ | USA (12 Indian Health Services Areas) | American Indian and Alaska Native | n/r | n/r | General population (USA) | ≥15 years | Registry | Crude |
| Ogden (1970)^129^ | USA (24 Reservation states west of Mississippi) | American Indian and Alaska Native | 630,000 | n/r | General population (USA) | All ages | Registry | Age adjusted (1940 US resident population) |
| Sievers (1975)^130^ | USA (Arizona) | Apache, Akimel O'odham, and other American Indian tribes | 40,361 | n/r | n/r | ≥10 years | Linked | Crude |
| Conrad (1974)^131^ | USA (Arizona) | Tohono O'odham | 12,179 | Indian Health Service Registry | n/r | All ages | Linked | Age adjusted (n/r) |
| Copeland (1989)^132^ | USA (Florida) | American Indian | 11,050 | Coroner/Medical Examiner records | General population (Dade County, Florida) | All ages | Medico–legal investigation records or database | Crude |
| Sievers (1990)^133^ | USA (Gila River Indian Community, Arizona) | Akimel O'odham | 4,915 | Death Certificate | General population (USA) | All ages | Linked | Age and sex adjusted (1980 US Population) |
| Kalish (1968)^134^ | USA (Hawai‘i) | Kānaka Maoli (Native Hawaiian) | n/r | n/r | White population (Hawai‘i, USA) | ≥14 years | Linked | Crude |
| Herne (2014)^135^ | USA (all Indian Health Service Areas) | American Indian and Alaska Native | n/r | Death certificate and/or linkage between Indian Health Service registration and National Death Index | White populations (USA by region) | All ages | Registry | Age adjusted (2000 US Standard Population) |
| Lester (1995)^136^ | USA (Lower 48 states) | American Indian and Alaska Native | Range by state: 984 – 166,464 | n/r | White population (USA) | All ages | Registry | Crude |
| Broudy (1983)^137^ | USA (New Mexico and Arizona) | Navajo | 162,303 | Indian Health Service Registry | General population (USA) | All ages | Registry | Age adjusted (1940 US population) |
| Becker (1990)^138^ | USA (New Mexico) | American Indian | n/r | Death certificate | White population (USA) | ≥15 years | Registry | Age adjusted (US 1970 Standard Population) |
| Levy (1965)^139^ | USA (New Mexico) | Navajo | 87,000 | n/r | General population (New Mexico, USA) | ≥10 years | Linked | Crude |
| Van Winkle (1993)^6^ | USA (New Mexico) | Apache, Navajo, and Pueblo | 58,936 (Navajo) | Death Certificate | General population (USA) | ≥5 years | Linked | Age adjusted (1940 US resident population) |
| Martin (2010)^140^ | USA (North Carolina) | American Indian | n/r | Linked to administrative registry | White population (USA) | ≥10 years | Linked | Crude |
| Humphrey (1982)^141^ | USA (North Carolina) | Lumbee and Cherokee | n/r | n/r | Population of adjacent counties (North Carolina) | All ages | Medico–legal investigation records or database | Crude |
| Simpson (1983)^142^ | USA (North–eastern Arizona) | Hopi | 9,406 | n/r | General population (USA) | All ages | Linked | Crude |
| Levy (1987)^143^ | USA (Northern Arizona) | Hopi | 7,600 | Linked to administrative registry | Yavapai county (primarily non–Indigenous population) | All ages | Linked | Crude |
| Shore (1975)^144^ | USA (Pacific Northwest) | American Indian | 23,921 | n/r | n/r | All ages | Linked | Crude |
| Christensen (2013)^145^ | USA (South Dakota) | American Indian | 82,073 | Funeral directors consulted families and reported on death certificate | White population (South Dakota, USA) | All ages | Registry | Age adjusted (2000 US Standard Population) |
| Wissow (2001)^146^ | USA (Southwest) | American Indian | 12,000 | Death Certificate | Non–Indigenous population in neighbouring counties | All ages | Linked | Crude |
| Miller (1979)^147^ | USA (Southwest) | American Indian | n/r | n/r | General population | All ages | Registry | Crude |
| Mullany (2009)^148^ | USA (White Mountain Apache Reservation, Arizona) | White Mountain Apache | 15,500 | Local surveillance system with multiple data sources (informant, medical, police) | General population (USA) | ≥5 years | Linked | Age adjusted (2000 US Census) |
| n/r=Not reported. WHO=World Health Organization. NAO= Nenets Autonomous Okrug, Russia. NWT=Northwest Territories, Canada. Population *n* are estimates based on reported counts in each article unless otherwise specified; these may not reflect denominators used to calculate incidence. ^*^General terms such as Indigenous, Pacific Peoples, or First Nation, were used when a specific nation or tribe was not identifiable. Where possible, contemporary terms were used. ^†^ Assumed all age groups were included in rate estimate calculations unless otherwise specified. ^‡^Mortality data sources included: registry data such as death certificates or vital statistics databases; medico–legal records from a coroner or medical examiner investigation; hospital or police records; information based on interviews with key informants such as family members; multiple linked sources which variously included two or more specific sources. ^¶^Total population for Micronesia: 142,298. Specific state/territory populations: 13,772 (Palau); 10,139 (Yap); 44,000 (Chuuk); 28,879 (Pohnpei); 6,448 (Kosrae); and 39,060 (Marshall Islands). **^§^**State/territory populations: Papua New Guinea (4,216,100), Fiji (n/r), Vanuatu (164,100), FSM (105,700), Marshall Islands (54,700), Palau (16,500), American Samoa (54,800), French Polynesia (218,000), and Western Samoa (163,400). ^\|\|^Population was not reported in Silviken et al. (2009)^60^ but was reported in earlier study by the lead author (Silviken et al. 2006)^149^ that used the same mortality and population data sources. ^**^Reported in Hezel (1989).^53^ **^††^**Reported in Booth (1999).^54^ ^‡‡^Calculated Indigenous population based on info available in article: 20.6% of Brazil's Indigenous population of 896,917 live in this region/state. ^¶¶^In Canada, First Nations people registered under the *Indian Act* are referred to “Status Indians” or “Registered Indians” by the federal government.^150^ **^§§^**Included “on reserve” First Nation populations in 7 Canadian provinces: Prince Edward Island, Nova Scotia, Quebec, Ontario, Manitoba, Saskatchewan, and Alberta. ^\|\|\|\|^By Indigenous group and territory: 5,284 (NWT, First Nation), 7,949 (NWT, Inuit), 2,207 (Yukon, First Nation). | | | | | | | | |
| ***Supplemental Table 1:* Characteristics of included studies** | | | | | | | | |

1. **Supplemental Table 2: Quality assessments with modified Newcastle-Ottawa Scale**

| **First Author (Year)** | **NOS** |
| --- | --- |
| **International, n=4** |  |
| Stevenson (1998)^51^ | 4 |
| Bramley (2004)^52^ | 4 |
| Hezel (1989)^53^ | 1 |
| Booth (1999)^54^ | 3 |
| **European Region, n=8** |  |
| Soininen (2008)^55^ | 3 |
| Bjerregaard (2015)^56^ | 2 |
| Thorslund (1989)^57^ | 3 |
| Bjorksten (2005)^58^ | 3 |
| Klomek (2016)^59^ | 4 |
| Silviken (2009)^60^ | 3 |
| Sumarokov (2014)^61^ | 4 |
| Hassler (2005)^62^ | 3 |
| **Western Pacific Region (Oceania), n=22** | |
| Pridmore (2009)^63^ | 4 |
| Measey (2006)^64^ | 4 |
| Clayer (1991)^65^ | 3 |
| Campbell (2016)^66^ | 4 |
| De Leo (2011)^67^ | 4 |
| Cantor (1997)^68^ | 3 |
| Langley (2000)^69^ | 3 |
| Langley (1990)^70^ | 3 |
| Beautrais (2006)^71^ | 4 |
| Rubinstein (1983)^72^ | 2 |
| Hezel (1984)^73^ | 2 |
| Pridmore (1995)^74^ | 3 |
| Price (1975)^75^ | 2 |
| Haynes (1984)^76^ | 2 |
| Ree (1971)^77^ | 2 |
| Pridmore (1994)^78^ | 3 |
| Booth (2010)^79^ | 2 |
| Parker (1966)^80^ | 2 |
| Smith (1981)^81^ | 1 |
| Pridmore (1997)^82^ | 2 |
| Vivili (1999)^83^ | 1 |
| De Leo (2013)^84^ | 2 |
| **Western Pacific (East Asia), n=9** | |
| Telisinghe (2014)^85^ | 2 |
| Wang (1997)^86^ | 2 |
| Lu (2013)^87^ | 2 |
| Ali (2014)^88^ | 2 |
| Jollant (2014)^89^ | 1 |
| Hsieh (1994)^90^ | 2 |
| Cheng (1992)^91^ | 3 |
| Wen (2004)^92^ | 2 |
| Liu (2011)^93^ | 2 |
| **Region of the Americas (Brazil & Canada), n=20** | |
| Machado (2015)^94^ | 3 |
| Orellana (2016)^95^ | 4 |
| Coloma (2006)^96^ | 3 |
| Souza (2013)^97^ | 4 |
| Mao (1992)^98^ | 4 |
| Isaacs (1998)^99^ | 2 |
| Wotton (1985)^100^ | 3 |
| Hislop (1987)^101^ | 4 |
| Macaulay (2004)^102^ | 3 |
| Ross (1986)^103^ | 3 |
| Chandler (1998)^104^ | 3 |
| Young (1983)^105^ | 2 |
| Pollock (2016)^106^ | 3 |
| Fox (1984)^107^ | 2 |
| Garro (1988)^108^ | 3 |
| Spaulding (1985)^109^ | 2 |
| Mao (1986)^110^ | 4 |
| Malchy (1997)^111^ | 4 |
| Penney (2009)^112^ | 3 |
| Butler (1965)^113^ | 2 |
| **Region of the Americas (Alaska, USA), n=12** | |
| Marshall (1998)^114^ | 2 |
| Kraus (1979)^115^ | 2 |
| Kettl (1991)^116^ | 2 |
| Holck (2013)^117^ | 3 |
| Hlady (1988)^118^ | 3 |
| Forbes (1988)^119^ | 3 |
| Day (2009)^120^ | 3 |
| Day (2003)^121^ | 4 |
| Andon (1997)^122^ | 3 |
| Travis (1983)^123^ | 2 |
| Travis (1984)^124^ | 2 |
| Wexler (2012)^125^ | 3 |
| **Region of the Americas (USA), n=24** | |
| Lester (1994)^126^ | 2 |
| Howard (2014)^127^ | 3 |
| Young (1993)^128^ | 2 |
| Ogden (1970)^129^ | 3 |
| Sievers (1975)^130^ | 2 |
| Conrad (1974)^131^ | 4 |
| Copeland (1989)^132^ | 3 |
| Sievers (1990)^133^ | 4 |
| Kalish (1968)^134^ | 2 |
| Herne (2014)^135^ | 4 |
| Lester (1995)^136^ | 2 |
| Broudy (1983)^137^ | 4 |
| Becker (1990)^138^ | 4 |
| Levy (1965)^139^ | 2 |
| Van Winkle (1993)^6^ | 4 |
| Martin (2010)^140^ | 3 |
| Humphrey (1982)^141^ | 2 |
| Simpson (1983)^142^ | 2 |
| Levy (1987)^143^ | 3 |
| Shore (1975)^144^ | 2 |
| Christensen (2013)^145^ | 4 |
| Wissow (2001)^146^ | 3 |
| Miller (1979)^147^ | 2 |
| Mullany (2009)^148^ | 4 |
| ***Mean NOS Score*** | **2.79** |
| NOS=Newcastle-Ottawa Scale score | |
| ***Supplemental Table 2:* Quality assessments with modified Newcastle-Ottawa Scale** | |

1. **Supplemental Table 3: Gender-specific suicide mortality incidence rates by WHO region and country**

|  |  | |  | |  | |  | | |  | |  | |  |  |  | |  | | |  |  |
| --- | --- | --- | --- | --- | --- | --- | --- | --- | --- | --- | --- | --- | --- | --- | --- | --- | --- | --- | --- | --- | --- | --- |
|  |  | |  | |  | | **Women** | | | |  | | |  |  | **Men** | |  | | |  |  |
|  |  | |  | |  | | **Indigenous** | | | | **Comparison Population** | | | |  | **Indigenous** | | | | | **Comparison Population** | |
| **WHO Region** | **Country** | | **Indigenous population** | | **Period** | | **CSIR** | | **SSIR** | | **CSIR** | | **SSIR** | |  | **CSIR** | | | **SSIR** | | **CSIR** | **SSIR** |
|  |  | |  | |  | |  | |  | |  | |  | |  |  | | |  | |  |  |
| **EUROPEAN REGION** | | |  | |  | |  | |  | |  | |  | |  |  | | |  | |  |  |
| Bjorksten (2005)^58^ | Greenland (West) | | Kalaallit (Inuit) | | 1995 | | 46.0 | | - | | - | | - | |  | 124.0 | | | - | | - | - |
| Silviken (2009)^60^ | Norway (Northern) | | Sámi | | 1970-1998 | | 8.4 | | - | | - | | - | |  | 28.5 | | | - | | - | - |
| Hassler (2005)^62^ | Sweden | | All Sámi | | 1961-2000 | | 3.8 | | - | | - | | - | |  | 19.5 | | | - | | - | - |
|  |  | | Sámi (Non-herding) | | 1961-2000 | | 3.3 | | - | | - | | - | |  | 16.4 | | | - | | - | - |
|  |  | | Sámi (Reindeer herding) | | 1961-2000 | | 5.9 | | - | | - | | - | |  | 30.1 | | | - | | - | - |
| Sumarokov (2014)^61^ | Russia (NAO) | | Nenets | | 2002-2012 | | 29.0 | | - | | 12.7 | | - | |  | 138.1 | | | - | | 86.3 | - |
| Klomek (2016)^59^ | Israel | | Bedouin | | 1999-2011 | | 2.9 | | 2.0 | | 3.3 | | 3.2 | |  | 5.9 | | | 4.5 | | 12.9 | 13.0 |
| **WESTERN PACIFIC REGION** | | |  | |  | |  | |  | |  | |  | |  |  | | |  | |  |  |
| Clayer (1991)^65^ | Australia (South Australia) | | ATSI | | 1988 | | 27.3 | | - | | - | | 6.2 | |  | 172.4 | | | - | | 28.5 | - |
| Cantor (1997)^68^ | Australia (Queensland) | | ATSI | | 1990-1992 | | - | | 3.8 | | - | | 6.5 | |  | - | | | 30.7 | | - | 24.4 |
| De Leo (2011)^67^ | Australia (Queensland) | | ATSI | | 1994-2007 | | - | | 9.4 | | - | | 5.3 | |  | - | | | 45.8 | | - | 20.1 |
| Measey (2006)^64^ | Australia (NT) | | ATSI | | 1999 | | - | | 11.2 | | - | | 5.9 | |  | - | | | 66.3 | | - | 34.0 |
| Campbell (2016)^66^ | Australia (Kimberly) | | ATSI | | 2005-2014 | | - | | 39.5 | | - | | - | |  | - | | | 117.0 | | - | - |
| Rubenstein (1983)^72^ | FSM | | Micronesian | | 1976-1979 | | 4.5 | | - | | - | | - | |  | 49.5 | | | - | | - | - |
| Booth (1999)^54^ | Papua New Guinea | | Pacific peoples | | 1990 | | <1 | | - | | - | | - | |  | <1 | | | - | | - | - |
|  | Samoa | | Samoan | | 1981 | | 21.0 | | - | | - | | - | |  | 41.0 | | | - | | - | - |
|  | Vanuatu | | ni-Vanuatu | | 1990-1992 | | 2.0 | | - | | - | | - | |  | 3.0 | | | - | | - | - |
|  | American Samoa | | Samoan | | 1990-1991 | | 0.0 | | - | | - | | - | |  | 34.0 | | | - | | - | - |
|  | French Polynesia | | Polynesian | | 1988-1992 | | 5.0 | | - | | - | | - | |  | 12.0 | | | - | | - | - |
|  | Fiji | | iTaukei | | 1982-1983 | | 3.0 | | - | | - | | - | |  | 4.0 | | | - | | - | - |
| Haynes (1984)^76^ | Fiji (Macuata) | | iTaukei | | 1979-1982 | | 0.0 | | - | | 71.9 | | - | |  | 11.6 | | | - | | 61.0 | - |
| Price (1975)^75^ | Fiji | | iTaukei | | 1971-1972 | | 0.4 | | - | | 15.3 | | - | |  | 2.1 | | | - | | 14.9 | - |
| Beautrais (2006)^71^ | Aotearoa/New Zealand | | Māori | | 2002 | | - | | 5.9 | | - | | 4.8 | |  | - | | | 19.7 | | - | 15.6 |
| Jollant (2014)^89^ | Philippines | | Palawan | | 2002-2012 | | 70.0 | | - | | - | | - | |  | 193.0 | | | - | | - | - |
| Hsieh (1994)^90^ | Taiwan | | Indigenous peoples | | 1971-1990 | | 29.9 | | - | | 8.3 | | - | |  | 48.4 | | | - | | 11.8 | - |
|  |  | | Atayal | | 1971-1990 | | 41.2 | | - | | 8.3 | | - | |  | 71.0 | | | - | | 11.8 | - |
|  |  | | Bunun | | 1971-1990 | | 40.7 | | - | | 8.3 | | - | |  | 48.2 | | | - | | 11.8 | - |
|  |  | | Paiwan | | 1971-1990 | | 12.4 | | - | | 8.3 | | - | |  | 28.8 | | | - | | 11.8 | - |
| Wen (2004)^92^ |  | | Indigenous peoples | | 1998-2000 | | 12.5 | | - | | - | | - | |  | 28.6 | | | - | | - | - |
| **REGION OF THE AMERICAS** | | |  | |  | |  | |  | |  | |  | |  |  | | |  | |  |  |
| Souza (2013)^97^ | Brazil (Amazonas) | | Indigenous peoples | | 2006-2010 | | - | | 8.6 | | - | | 1.3 | |  | - | | | 28.2 | | - | 8.8 |
| Hislop (1987)^101^ | Canada (British Columbia) | | First Nation | | 1953-1978 | | - | | 13.1 | | - | | 8.0 | |  | - | | | 39.4 | | - | 20.7 |
| Mao (1986)^110^ | Canada (7 provinces) | | First Nation | | 1977-1982 | | - | | 17.0 | | - | | 6.4 | |  | - | | | 53.0 | | - | 19.9 |
| Mao (1992)^98^ | Canada | | First Nation | | 1981 | | - | | 19.4 | | - | | 6.6 | |  | - | | | 58.6 | | - | 21.5 |
| Ross (1986)^103^ | Canada (Manitoba) | | Cree | | 1981-1984 | | 24.0 | | - | | 0.0 | | - | |  | 142.0 | | | - | | 0.0 | - |
| Macaulay (2004)^102^ | Canada (Nunavut) | | Inuit | | 1987-1996 | | - | | 12.0 | | - | | - | |  | - | | | 74.0 | | - | - |
| Pollock (2016)^106^ | Canada (Labrador) | | Innu | | 1993-2009 | | 0.0 | | 0.0 | | 2.5 | | 2.4 | |  | 272.0 | | | 223.2 | | 14.2 | 14.0 |
|  |  | | Inuit | | 1993-2009 | | 97.1 | | 75.5 | | 2.5 | | 2.4 | |  | 272.6 | | | 248.7 | | 14.2 | 14.0 |
| Young (1993)^128^ | USA (All IHSAs) | | AI/AN | | 1979-1981 | | 4.8 | | - | | 5.7 | | - | |  | 33.1 | | | - | | 18.9 | - |
| Lester (1994)^126^ | USA | | AI/AN | | 1980 | | 4.9 | | - | | 6.1 | | - | |  | 22.0 | | | - | | 20.6 | - |
| Herne (2014)^135^ | USA (All IHSAs) | | AI/AN | | 1999-2009 | | - | | 8.7 | | - | | 5.9 | |  | - | | | 34.7 | | - | 23.2 |
|  | USA (Pacific Coast IHSA) | | AI/AN | | 1999-2009 | | - | | 8.4 | | - | | 6.3 | |  | - | | | 29.0 | | - | 24.3 |
|  | USA (Southwest IHSA) | | AI/AN | | 1999-2009 | | - | | 6.8 | | - | | 8.6 | |  | - | | | 33.9 | | - | 31.5 |
|  | USA (South Plains IHSA) | | AI/AN | | 1999-2009 | | - | | 6.9 | | - | | 6.3 | |  | - | | | 31.5 | | - | 21.3 |
|  | USA (North Plains IHSA) | | AI/AN | | 1999-2009 | | - | | 11.9 | | - | | 4.6 | |  | - | | | 41.6 | | - | 21.0 |
|  | USA (East IHSA) | | AI/AN | | 1999-2009 | | - | | 4.1 | | - | | 4.8 | |  | - | | | 13.0 | | - | 18.9 |
|  | USA (Alaska IHSA) | | AI/AN | | 1999-2009 | | - | | 19.3 | | - | | 6.7 | |  | - | | | 65.4 | | - | 27.9 |
| Forbes (1988)^119^ | USA (Alaska) | | Alaska Native | | 1985 | | 24.1 | | - | | 6.8 | | - | |  | 100.1 | | | - | | 24.1 | - |
| Kettl (1991)^116^ | USA (Alaska) | | Alaska Native | | 1979-1984 | | 8.5 | | - | | 6.1 | | - | |  | 38.2 | | | - | | 20.7 | - |
| Day (2003)^121^ | USA (Alaska) | | Alaska Native | | 1989-1998 | | - | | 17.1 | | - | | 4.6 | |  | - | | | 82.4 | | - | 19.4 |
| Day (2009)^120^ | USA (Alaska) | | Alaska Native | | 1999-2003 | | - | | 15.4 | | - | | 6.6 | |  | - | | | 56.7 | | - | 27.1 |
| Holck (2013)^117^ | USA (Alaska) | | Alaska Native | | 2004-2008 | | - | | 21.4 | | - | | 5.0 | |  | - | | | 62.8 | | - | 19.6 |
| Becker (1990)^138^ | USA (New Mexico) | | American Indian | | 1983-1987 | | - | | 2.7 | | - | | 5.0 | |  | - | | | 39.9 | | - | 19.7 |
| Kalish (1968)^134^ | USA (Hawai‘i) | | Native Hawaiian | | 1959-1965 | | 10.2 | | - | | 8.3 | | - | |  | 24.8 | | | - | | 15.2 | - |
|  |  | | Other Pacific peoples | | 1959-1965 | | 4.0 | | - | | 8.3 | | - | |  | 9.7 | | | - | | 15.2 | - |
|  |  | |  | |  | |  | |  | |  | |  | |  |  | | |  | |  |  |
| WHO=World Health Organization. CSIR=Crude Suicide Incidence Rate. SSIR=Standardized Suicide Incidence Rate. NAO=Nenets Autonomous Okrug. NT=Northern Territory. ATSI=Aboriginal and Torres Strait Islander. FSM=Federated States of Micronesia. IHSA=Indian Health Service Area. AI/AN=American Indian and Alaska Native. | | | | | | | | | | | | | | | | | | | | | | |
|  | |  | |  | |  | |  | |  | |  | |  |  | |  |  | |  | |  |
| ***Supplemental Table 3:* Gender-specific suicide mortality incidence rates by WHO region and country** | | | | | | | | | | |  | | |  |  | |  |  | |  | |  |

1. **Supplemental Table 4: Incidence rate ratios by WHO region and country**

| **First Author (Year)** | **Indigenous peoples, tribe, or group (Region)** | **IRR** |
| --- | --- | --- |
| **EUROPEAN REGION** | |  |
| Soininen (2008)^55^ | Sámi (Northern Finland) | 1.90 |
| Silviken (2009)^60^ | Sámi (Northern Norway) | 1.45 |
| Sumarokov (2014)^61^ | Nenets (Nenets Autonomous Okrug, Russia) | 1.43 |
| Klomek (2016)^59^ | Bedouin (Israel) | 0.40 |
| **WESTERN PACIFIC REGION (Australia)** | |  |
| Campbell (2016)^66^ | Aboriginal and Torres Strait Islanders (Kimberly) | 7.40 |
| Clayer (1991)^65^ | Aboriginal and Torres Strait Islanders (South Australia) | 6.05 |
| Pridmore (2009)^63^ | Aboriginal and Torres Strait Islanders (Northern Territory) | 2.50 |
| Measey (2006)^64^ | Aboriginal and Torres Strait Islanders (Northern Territory) | 2.21 |
| De Leo (2011)^67^ | Aboriginal and Torres Strait Islanders (Queensland) | 2.16 |
| Bramley (2004)^52^ | Aboriginal and Torres Strait Islander | 1.60 |
| Cantor (1997)^68^ | Aboriginal and Torres Strait Islanders (Queensland) | 1.11 |
| Stevenson (1998)^51^ | Aboriginal and Torres Strait Islanders | 0.90 |
| **WESTERN PACIFIC REGION (Oceania)** | |  |
| Booth (2010)^79^ | Chamorro (Guam) | 3.00 |
| Bramley (2004)^52^ | Māori (Aotearoa/New Zealand) | 1.00 |
| Langley (1990)^70^ | Māori (Aotearoa/New Zealand) | 0.67 |
| Parker (1966)^80^ | Pacific peoples (Papua New Guinea) | 0.05 |
| Ree (1971)^77^ | iTaukei (Macuata, Fiji) | 0.17 |
| Pridmore (1995)^74^ | iTaukei (Fiji) | 0.16 |
| Pridmore (1994)^78^ | iTaukei (Western Division, Fiji) | 0.11 |
| Booth (1999)^54^ | iTaukei (Fiji) | 0.09 |
| Haynes (1984)^76^ | iTaukei (Macuata, Fiji) | 0.09 |
| Price (1975)^75^ | iTaukei (Fiji) | 0.09 |
| **WESTERN PACIFIC REGION (East Asia)** | |  |
| Hsieh (1994)^90^ | Atayal (Taiwan) | 5.69 |
| Cheng (1992)^91^ | Bunun (Taiwan) | 5.54 |
| Hsieh (1994)^90^ | Bunun (Taiwan) | 4.42 |
| Hsieh (1994)^90^ | Atayal, Bunun, and Paiwan (Taiwan) | 3.96 |
| Cheng (1992)^91^ | Atayal (Taiwan) | 3.96 |
| Liu (2011)^93^ | Atayal (East Taiwan) | 3.79 |
| Hsieh (1994)^90^ | Paiwan (Taiwan) | 2.11 |
| Cheng (1992)^91^ | Paiwan (Taiwan) | 1.39 |
| Liu (2011)^93^ | Ami (East Taiwan) | 0.87 |
| Cheng (1992)^91^ | Ami (Taiwan) | 0.45 |
| Telisinghe (2014)^85^ | 7 tribes (Brunei Darussalam) | 3.40 |
| Jollant (2014)^89^ | Palawan (Philippines) | 2.48 |
| Ali (2014)^88^ | Bumiputera (Sabah and Sarawak, Malaysia) | 1.16 |
| Lu (2013)^87^ | Li su (Yunnan Province, China) | 2.33 |
|  | Yi (Yunnan Province, China) | 0.95 |
|  | Dai (Yunnan Province, China) | 0.55 |
|  | 10 ethnic minority groups* | 0.04-1.67 |
| Wang (1997)^86^ | Meng (Hohhot, Inner Mongolia, China) | 0.54 |
| Wang (1997)^86^ | Hui (Hohhot, Inner Mongolia, China) | 0.27 |
| **REGION OF THE AMERICAS (Brazil and Canada)** | |  |
| Coloma (2006)^96^ | 6 tribes (Mato Grosso do Sul) | 20.04 |
| Souza (2013)^97^ | Indigenous peoples (Tabatinga, Amazonas) | 18.04 |
| Souza (2013)^97^ | Indigenous peoples (Sao Gabriel da Cachoeira, Amazonas) | 9.98 |
| Orellana (2016)^95^ | 3 tribes (Mato Grosso do Sul) | 8.10 |
| Souza (2013)^97^ | Indigenous peoples (Amazonas) | 4.38 |
| Machado (2015)^94^ | Indigenous peoples | 2.18 |
| Souza (2013)^97^ | Indigenous peoples (Manaus, Amazonas) | 0.00 |
| Pollock (2016)^106^ | Inuit (Labrador) | 20.60 |
| Penney (2009)^112^ | Inuit (Nunavik, Quebec) | 16.00 |
| Pollock (2016)^106^ | Innu (Labrador) | 14.20 |
| Penney (2009)^112^ | Inuit (Nunavut) | 9.60 |
| Isaacs (1998)^99^ | Inuit (NWT/Nunavut) | 5.26 |
| Wotton (1985)^100^ | Innu and Inuit (Labrador) | 4.58 |
| Butler (1965)^113^ | First Nation (Yukon) | 4.49 |
| Macaulay (2004)^102^ | Inuit (Killaviq, Nunavut) | 3.47 |
| Chandler (1998)^104^ | First Nation (British Columbia) | 3.05 |
| Malchy (1997)^111^ | First Nation and Métis (Manitoba) | 2.30 |
| Bramley (2004)^52^ | First Nations | 2.30 |
| Isaacs (1998)^99^ | Dene (NWT) | 1.93 |
| Butler (1965)^113^ | Inuit (NWT/Nunavut) | 0.94 |
| **REGION OF THE AMERICAS (USA, National and Regional)** | |  |
| Herne (2014)^135^ | American Indian and Alaska Native (Alaska IHSA) | 2.45 |
|  | American Indian and Alaska Native (North Plains IHSA) | 2.09 |
|  | American Indian and Alaska Native (Pacific Coast IHSA) | 1.22 |
|  | American Indian and Alaska Native (South Plains IHSA) | 1.21 |
|  | American Indian and Alaska Native (Southwest IHSA) | 1.01 |
|  | American Indian and Alaska Native (East IHSA) | 0.73 |
|  | American Indian and Alaska Native (All IHSAs) | 1.49 |
| Ogden (1970)^129^ | American Indian and Alaska Native (24 western states) | 2.10 |
| Young (1993)^128^ | American Indian and Alaska Native | 1.63 |
| Stevenson (1998)^51^ | American Indian | 1.40 |
| Bramley (2004)^52^ | American Indian and Alaska Native | 1.20 |
| Howard (2014)^127^ | American Indian and Alaska Native | 1.07 |
| Lester (1994)^126^ | American Indian and Alaska Native | 1.01 |
| **REGION OF THE AMERICAS (USA, Alaska)** | |  |
| Marshall (1998)^114^ | Athabascan | 7.00 |
| Marshall (1998)^114^ | Inupiat | 4.24 |
| Andon (1997)^122^ | Athabascan | 4.24 |
| Day (2003)^121^ | Alaska Native | 4.20 |
| Forbes (1988)^119^ | Alaska Native | 4.14 |
| Holck (2013)^117^ | Alaska Native | 3.53 |
| Marshall (1998)^114^ | Yup'ik | 2.52 |
| Marshall (1998)^114^ | Yup'ik, Inupiat, and Athabascan | 2.33 |
| Kraus (1979)^115^ | Alaska Native | 2.24 |
| Hlady (1988)^118^ | Alaska Native | 2.20 |
| Day (2009)^120^ | Alaska Native | 2.20 |
| Kettl (1991)^116^ | Alaska Native | 1.92 |
| **REGION OF THE AMERICAS (USA, Lower 48 States and Hawai‘i)** | |  |
| Sievers (1990)^133^ | Akimel O'odham (Arizona) | 4.30 |
| Van Winkle (1993)^6^ | Apache (New Mexico) | 4.20 |
| Mullany (2009)^148^ | White Mountain Apache (Arizona) | 3.70 |
| Humphrey (1982)^141^ | Cherokee (North Carolina) | 3.14 |
| Van Winkle (1993)^6^ | Pueblo (New Mexico) | 2.80 |
| Miller (1979)^147^ | American Indian (Southwest USA) | 2.69 |
| Broudy (1983)^137^ | American Indian (Arizona and New Mexico) | 2.30 |
| Christensen (2013)^145^ | American Indian (South Dakota) | 2.11 |
| Simpson (1983)^142^ | Hopi (Arizona) | 2.08 |
| Van Winkle (1993)^6^ | Navajo (New Mexico) | 1.60 |
| Kalish (1968)^134^ | Kānaka Maoli (Native Hawaiian) | 1.48 |
| Wissow (2001)^146^ | American Indian (Southwest) | 1.35 |
| Levy (1987)^143^ | American Indian (Arizona) | 1.04 |
| Humphrey (1982)^141^ | Lumbee (North Carolina) | 0.70 |
| Levy (1965)^139^ | Navajo (New Mexico) | 0.72 |
| Martin (2010)^140^ | American Indian (North Carolina) | 0.59 |
| Copeland (1989)^132^ | American Indian (Florida) | 0.58 |
| ***Supplemental Table 4*: Incidence rate ratios by WHO region and country** | | |

**Additional File 1: Supplement 4 (PRISMA CHECKLIST^14^)**

| **Section/topic** | **#** | **Checklist item** | **Reported on page #** |
| --- | --- | --- | --- |
| **TITLE** Global incidence of suicide among Indigenous Peoples: a systematic review | | |  |
| Title | 1 | Identify the report as a systematic review, meta-analysis, or both. | 1 |
| **ABSTRACT** | | |  |
| Structured summary | 2 | Provide a structured summary including, as applicable: background; objectives; data sources; study eligibility criteria, participants, and interventions; study appraisal and synthesis methods; results; limitations; conclusions and implications of key findings; systematic review registration number. | 2 |
| **INTRODUCTION** | | |  |
| Rationale | 3 | Describe the rationale for the review in the context of what is already known. | 3-4 |
| Objectives | 4 | Provide an explicit statement of questions being addressed with reference to participants, interventions, comparisons, outcomes, and study design (PICOS). | 3-4 |
| **METHODS** | | |  |
| Protocol and registration | 5 | Indicate if a review protocol exists, if and where it can be accessed (e.g., Web address), and, if available, provide registration information including registration number. | Supplement 1 |
| Eligibility criteria | 6 | Specify study characteristics (e.g., PICOS, length of follow-up) and report characteristics (e.g., years considered, language, publication status) used as criteria for eligibility, giving rationale. | 5 + Supplement 2 |
| Information sources | 7 | Describe all information sources (e.g., databases with dates of coverage, contact with study authors to identify additional studies) in the search and date last searched. | 5 + Supplement 2 |
| Search | 8 | Present full electronic search strategy for at least one database, including any limits used, such that it could be repeated. | 5 + Supplement 2 |
| Study selection | 9 | State the process for selecting studies (i.e., screening, eligibility, included in systematic review, and, if applicable, included in the meta-analysis). | 6 + Supplement 2 |
| Data collection process | 10 | Describe method of data extraction from reports (e.g., piloted forms, independently, in duplicate) and any processes for obtaining and confirming data from investigators. | 6 |
| Data items | 11 | List and define all variables for which data were sought (e.g., PICOS, funding sources) and any assumptions and simplifications made. | 6 + Supplement 2 |
| Risk of bias in individual studies | 12 | Describe methods used for assessing risk of bias of individual studies (including specification of whether this was done at the study or outcome level), and how this information is to be used in any data synthesis. | 7 + Supplement 2 |
| Summary measures | 13 | State the principal summary measures (e.g., risk ratio, difference in means). | 6 |
| Synthesis of results | 14 | Describe the methods of handling data and combining results of studies, if done, including measures of consistency (e.g., I^2^) for each meta-analysis. | 6 |

| **Section/topic** | **#** | **Checklist item** | **Reported on page #** |
| --- | --- | --- | --- |
| Risk of bias across studies | 15 | Specify any assessment of risk of bias that may affect the cumulative evidence (e.g., publication bias, selective reporting within studies). | 7-8 |
| Additional analyses | 16 | Describe methods of additional analyses (e.g., sensitivity or subgroup analyses, meta-regression), if done, indicating which were pre-specified. | n/a |
| **RESULTS** | | |  |
| Study selection | 17 | Give numbers of studies screened, assessed for eligibility, and included in the review, with reasons for exclusions at each stage, ideally with a flow diagram. | 8 + Figure 1 |
| Study characteristics | 18 | For each study, present characteristics for which data were extracted (e.g., study size, PICOS, follow-up period) and provide the citations. | 12-16 + Supplement |
| Risk of bias within studies | 19 | Present data on risk of bias of each study and, if available, any outcome level assessment (see item 12). | Supplement |
| Results of individual studies | 20 | For all outcomes considered (benefits or harms), present, for each study: (a) simple summary data for each intervention group (b) effect estimates and confidence intervals, ideally with a forest plot. | 7-9 + Supplement 3 |
| Synthesis of results | 21 | Present results of each meta-analysis done, including confidence intervals and measures of consistency. | n/a |
| Risk of bias across studies | 22 | Present results of any assessment of risk of bias across studies (see Item 15). | 12-13 + Supplement |
| Additional analysis | 23 | Give results of additional analyses, if done (e.g., sensitivity or subgroup analyses, meta-regression [see Item 16]). | n/a |
| **DISCUSSION** | | |  |
| Summary of evidence | 24 | Summarize the main findings including the strength of evidence for each main outcome; consider their relevance to key groups (e.g., healthcare providers, users, and policy makers). | 9-11 |
| Limitations | 25 | Discuss limitations at study and outcome level (e.g., risk of bias), and at review-level (e.g., incomplete retrieval of identified research, reporting bias). | 12-13 |
| Conclusions | 26 | Provide a general interpretation of the results in the context of other evidence, and implications for future research. | 9-10 |
| **FUNDING** | | |  |
| Funding | 27 | Describe sources of funding for the systematic review and other support (e.g., supply of data); role of funders for the systematic review. | 7, 15 |

**References**

1. Gracey M, King M. Indigenous health part 1: determinants and disease patterns. *The Lancet* 2009; **374**(9683): 65-75.

2. United Nations Department of Economic and Social Affairs. State of the World's Indigenous Peoples. United Nations; 2009. http://www.un.org/esa/socdev/unpfii/documents/SOWIP/en/SOWIP_web.pdf (accessed November 18, 2017).

3. Hunter E, Harvey D. Indigenous suicide in Australia, New Zealand, Canada and the United States. *Emergency Medicine* 2002; **14**(1): 14-23.

4. Kirmayer LJ, Brass GM, Holton T, Paul K, Simpson C, Tait CL. Suicide among Aboriginal People in Canada. Ottawa, Canada: Aboriginal Healing Foundation; 2007.

5. Wexler L, Silveira ML, Bertone-Johnson E. Factors associated with Alaska Native fatal and nonfatal suicidal behaviors 2001-2009: trends and implications for prevention. *Archives of Suicide Research* 2012; **16**(4): 273-86.

6. Van Winkle N, May PA. An update on American Indian suicide in New Mexico, 1980-1987. *Human Organization* 1993; **52**(3): 304-15.

7. Harder HG, Rash J, Holyk T, Jovel E, Harder K. Indigenous youth suicide: a systematic review of the literature. *Pimatisiwin: A Journal of Aboriginal and Indigenous Community Health* 2012; **10**(1): 125-42.

8. Morris P, Maniam T. Suicide in Fiji: a review of the literature. *Asia Pacific Journal of Public Health* 2001; **12**(1): 46-9.

9. Ran M. Suicide in Micronesia: a systematic review. *Primary Psychiatry* 2007; **14**(11): 80.

10. Alcántara C, Gone JP. Reviewing suicide in Native American communities: Situating risk and protective factors within a transactional–ecological framework. *Death Studies* 2007; **31**(5): 457-77.

11. Lehti V, Niemelä S, Hoven C, Mandell D, Sourander A. Mental health, substance use and suicidal behaviour among young Indigenous people in the Arctic: a systematic review. *Social Science & Medicine* 2009; **69**(8): 1194-203.

12. Clarke VA, Frankish CJ, Green LW. Understanding suicide among Indigenous adolescents: a review using the PRECEDE model. *Injury Prevention* 1997; **3**(2): 126-34.

13. Allen J, Levintova M, Mohatt G. Suicide and alcohol-related disorders in the U.S. Arctic: boosting research to address a primary determinant of health disparities. *International Journal of Circumpolar Health* 2011; **70**(5): 473-87.

14. Moher D, Liberati A, Tetzlaff J, Altman DG, The PG. Preferred Reporting Items for Systematic Reviews and Meta-Analyses: The PRISMA Statement. *PLOS Medicine* 2009; **6**(7): e1000097.

15. Tollefson D, Bloss E, Fanning A, Redd J, Barker K, McCray E. Burden of tuberculosis in Indigenous peoples globally: a systematic review. *The International Journal of Tuberculosis and Lung Disease* 2013; **17**(9): 1139-50.

16. Arnold M, Moore SP, Hassler S, Ellison-Loschmann L, Forman D, Bray F. The burden of stomach cancer in Indigenous populations: a systematic review and global assessment. *Gut* 2013; **63**: 64-71.

17. Bartlett JG, Madariaga-Vignudo L, Neil JD, Kuhnlein HV. Identifying Indigenous peoples for health research in a global context: a review of perspectives and challenges. *International Journal of Circumpolar Health* 2007; **66**(4).

18. Valery PC, Moore SP, Meiklejohn J, Bray F. International variations in childhood cancer in Indigenous populations: a systematic review. *The Lancet Oncology* 2014; **15**(2): e90-e103.

19. Naqshbandi M, Harris SB, Esler JG, Antwi-Nsiah F. Global complication rates of type 2 diabetes in Indigenous peoples: a comprehensive review. *Diabetes Research and Clinical Practice* 2008; **82**(1): 1-17.

20. Kirmayer LJ. Suicide among Canadian Aboriginal peoples. *Transcultural Psychiatry* 1994; **31**(1): 3-58.

21. Kolahdooz F, Jang SL, Corriveau A, Gotay C, Johnston N, Sharma S. Knowledge, attitudes, and behaviours towards cancer screening in Indigenous populations: a systematic review. *The Lancet Oncology* 2014; **15**(11): e504-e16.

22. Wells G, Shea B, O’Connell D, et al. The Newcastle-Ottawa Scale (NOS) for assessing the quality of nonrandomised studies in meta-analyses. 2000. http://www.ohri.ca/programs/clinical_epidemiology/oxford.asp (accessed November 17, 2017).

23. Armitage CJ, Panagioti M, Abdul Rahim W, Rowe R, O'Connor RC. Completed suicides and self-harm in Malaysia: a systematic review. *General hospital psychiatry* 2015; **37**(2): 153-65.

24. Azuero AJ, Arreaza-Kaufman D, Coriat J, et al. Suicide in the Indigenous population of Latin America: a systematic review. *Revista Colombiana de Psiquiatría* 2017.

25. Berlin IN. Suicide among American Indian adolescents: an overview. *Suicide and Life-Threatening Behavior* 1987; **17**(3): 218-32.

26. Clifford AC, Doran CM, Tsey K. A systematic review of suicide prevention interventions targeting Indigenous peoples in Australia, United States, Canada and New Zealand. *BMC Public Health* 2013; **13**(1): 463.

27. Else IRN, Andrade NN, Nahulu LB. Suicide and suicidal-related behaviors among indigenous Pacific Islanders in the United States. *Death Studies* 2007; **31**(5): 479-501.

28. Harlow AF, Bohanna I, Clough A. A systematic review of evaluated suicide prevention programs targeting Indigenous youth. *Crisis* 2014; **35**(5): 310-21.

29. Jordans M, Kaufman A, Brenman N, et al. Suicide in South Asia: a scoping review. *BMC Psychiatry* 2014; **14**(1): 358.

30. Leenaars AA. Suicide among indigenous peoples: introduction and call to action. *Archives of Suicide Research* 2006; **10**(2): 103-15.

31. Leenaars AA, EchoHawk M, Lester D, Leenaars L. Suicide among Indigenous Peoples: what does the international knowledge tell us? *Canadian Journal of Native Studies* 2007; **27**(2): 479.

32. Lester D. Suicide among indigenous peoples: a cross-cultural perspective. *Archives of Suicide Research* 2006; **10**(2): 117-24.

33. MacNeil MS. An epidemiologic study of Aboriginal adolescent risk in Canada: the meaning of suicide. *Journal of Child and Adolescent Psychiatric Nursing* 2008; **21**(1): 3-12.

34. Mars B, Burrows S, Hjelmeland H, Gunnell D. Suicidal behaviour across the African continent: a review of the literature. *BMC Public Health* 2014; **14**(1): 606.

35. May PA. A bibliography on suicide and suicide attempts among American Indians and Alaska Natives. *Omega: Journal of Death and Dying* 1990; **21**(3): 199-214.

36. McIntosh JL, Santos JF. Suicide among Native Americans: a compilation of findings. *Omega: Journal of Death and Dying* 1980; **11**(4): 303-16.

37. McIntosh JL. Suicide among Native Americans: Further Tribal Data and Considerations. *Omega: Journal of Death and Dying* 1984; **14**(3): 215-29.

38. McLoughlin A, Gould M, Malone K. Global trends in teenage suicide: 2003-2014. *QJM: Monthly journal of the Association of Physicians* 2015; **108**(10): 765-80.

39. Middlebrook DL, LeMaster PL, Beals J, Novins DK, Manson SM. Suicide prevention in American Indian and Alaska Native communities: a critical review of programs. *Suicide and Life-Threatening Behavior* 2001; **31 Suppl**: 132-49.

40. Olson LM, Wahab S. American Indians and suicide: a neglected area of research. *Trauma, Violence, and Abuse* 2006; **7**(1): 19-33.

41. Owens MV. Bibliography of Health Issues Affecting North American Indians, Eskimos, and Aleuts: 1950-1988. Rockville, MD: Indian Health Service (PSA/HSA), 1990.

42. Peters R. Suicidal behavior among Native Americans: an annotated bibliography. *White Cloud Journal of American Indian/Alaska Native Mental Health* 1981; **2**(3): 9-20.

43. Redvers J, Bjerregaard P, Eriksen H, et al. A scoping review of Indigenous suicide prevention in Circumpolar regions. *International Journal of Circumpolar Health* 2015.

44. Reser JP. Indigenous suicide in cross-cultural context: an overview statement and selective bibliography of sources relevant to Indigenous suicide in Australia, North America, and the Pacific. *South Pacific Journal of Psychology* 1999; **11**(2): 95-111.

45. Santora D, Starkey P. Research studies in American Indian suicides. *Journal of Psychosocial Nursing and Mental Health Services* 1982; **20**(8): 25-9.

46. Strickland CJ. Suicide among American Indian, Alaskan Native, and Canadian Aboriginal youth: advancing the research agenda. *International Journal of Mental Health* 1996; **25**(4): 11-32.

47. Thompson JW, Walker RD. Adolescent suicide among American Indians and Alaska Natives. *Psychiatric Annals* 1990; **20**(3): 128-33.

48. Tousignant M. Suicide in small-scale societies. *Transcultural Psychiatry* 1998; **35**(2): 291-306.

49. Wyatt LC, Ung T, Park R, Kwon SC, Trinh-Shevrin C. Risk factors of suicide and depression among Asian American, Native Hawaiian, and Pacific Islander youth: a systematic literature review. *Journal of Health Care for the Poor and Underserved* 2015; **26**(2 0): 191-237.

50. Young TK, Revich B, Soininen L. Suicide in Circumpolar regions: an introduction and overview. *International Journal of Circumpolar Health* 2015; **74**: 27349.

51. Stevenson MR, Wallace LJD, Harrison J, Moller J, Smith RJ. At risk in two worlds: injury mortality among Indigenous people in the US and Australia, 1990-92. *Australian and New Zealand Journal of Public Health* 1998; **22**(6): 641-4.

52. Bramley D, Hebert P, Jackson R, Chassin M. Indigenous disparities in disease-specific mortality, a cross-country comparison: New Zealand, Australia, Canada, and the United States. *New Zealand Medical Journal* 2004; **117**(1207): U1215.

53. Hezel FX. Suicide and the Micronesian family. *The Contemporary Pacific* 1989: 43-74.

54. Booth H. Pacific Island suicide in comparative perspective. *Journal of Biosocial Science* 1999; **31**(4): 433-48.

55. Soininen L, Pukkala E. Mortality of the Sámi in northern Finland 1979-2005. *International Journal of Circumpolar Health* 2008; **67**(1).

56. Bjerregaard P, Larsen CVL. Time trend by region of suicides and suicidal thoughts among Greenland Inuit. *International Journal of Circumpolar Health* 2015; **74**: 10.3402/ijch.v74.26053.

57. Thorslund J, Misfeldt J. On suicide statistics. *Arctic Medical Research* 1989; **48**(3): 124-30.

58. Bjorksten KS, Bjerregaard P, Kripke DF. Suicides in the midnight sun - A study of seasonality in suicides in West Greenland. *Psychiatry Research* 2005; **133**(2-3): 205-13.

59. Klomek AB, Nakash O, Goldberger N, et al. Completed suicide and suicide attempts in the Arab population in Israel. *Social Psychiatry and Psychiatric Epidemiology* 2016; **51**(6): 869-76.

60. Silviken A. Prevalence of suicidal behaviour among indigenous Sámi in northern Norway. *International Journal of Circumpolar Health* 2009; **68**(3): 204-11.

61. Sumarokov YA, Brenn T, Kudryavtsev AV, Nilssen O. Suicides in the Indigenous and non-Indigenous populations in the Nenets Autonomous Okrug, northwestern Russia, and associated socio-demographic characteristics. *International Journal of Circumpolar Health* 2014; **73**: 24308.

62. Hassler S, Johansson R, Sjölander P, Grönberg H, Damber L. Causes of death in the Sámi population of Sweden, 1961–2000. *International Journal of Epidemiology* 2005; **34**(3): 623-9.

63. Pridmore S, Fujiyama H. Suicide in the Northern Territory, 2001-2006. *The Australian and New Zealand Journal of Psychiatry* 2009; **43**(12): 1126-30.

64. Measey MAL, Li SQ, Parker R, Wang Z. Suicide in the Northern Territory, 1981-2002. *Medical Journal of Australia* 2006; **185**(6): 315-9.

65. Clayer JR, Czechowicz AS. Suicide with Aboriginal people in South Australia: comparison with suicide deaths in the total urban and rural populations. *Medical Journal of Australia* 1991; **154**(10): 683-5.

66. Campbell A, Chapman M, McHugh C, Sng A, Balaratnasingam S. Rising Indigenous suicide rates in Kimberley and implications for suicide prevention. *Australasian Psychiatry* 2016; **24**(6): 561-4.

67. De Leo D, Sveticic J, Milner A. Suicide in Indigenous people in Queensland, Australia: trends and methods, 1994 – 2007. *The Australian and New Zealand Journal of Psychiatry* 2011; **45**(7): 532-8.

68. Cantor CH, Slater PJ. A regional profile of suicide in Queensland. *Australian and New Zealand Journal of Public Health* 1997; **21**(2): 181-6.

69. Langley J, Broughton J. Injury to Maori I: Fatalities. *New Zealand Medical Journal* 2000; **113**(1123): 508-10.

70. Langley JD, Johnston SE. Purposely self-inflicted injury resulting in death and hospitalisation in New Zealand. *Community Health Studies* 1990; **14**(2): 190-9.

71. Beautrais AL, Fergusson DM. Indigenous suicide in New Zealand. *Archives of Suicide Research* 2006; **10**(2): 159-68.

72. Rubinstein DH. Epidemic suicide among Micronesian adolescents. *Social Science & Medicine* 1983; **17**(10): 657-65.

73. Hezel FX. Cultural patterns in Trukese suicide. *Ethnology* 1984; **23**(3): 193-206.

74. Pridmore S, Ryan K, Blizzard L. Victims of violence in Fiji. *The Australian and New Zealand Journal of Psychiatry* 1995; **29**(4): 666-70.

75. Price J, Karim I. Suicide in Fiji: a two year survey. *Acta Psychiatrica Scandinavica* 1975; **52**(3): 153-9.

76. Haynes RH. Suicide in Fiji: a preliminary study. *British Journal of Psychiatry* 1984; **145**: 433-8.

77. Ree GH. Suicide in Macuata province, Fiji: a review of 73 cases. *The Practitioner* 1971; **207**(241): 669-71.

78. Pridmore S, Ryan K. The influence of race and sex on the method of suicide in the western division of Fiji. *Fiji Medical Journal* 1994; **20**: 9-12.

79. Booth H. The evolution of epidemic suicide on Guam: context and contagion. *Suicide and Life-Threatening Behavior* 2010; **40**(1): 1-13.

80. Parker N, Burton-Bradley B. Suicide in Papua and New Guinea. *Medical Journal of Australia* 1966; **2**(24): 1125.

81. Smith D. Suicide in a remote preliterate society in the highlands of Papua New Guinea. *Papua and New Guinea medical journal* 1981; **24**(4): 242-6.

82. Pridmore S. Suicidal behavior in the Honiara area of the Solomon Islands. *International Journal of Mental Health* 1997; **25**(4): 33-8.

83. Vivili P, Finau S, Finau E. Suicide in Tonga, 1982-1997. *Pacific Health Dialog: Journal of Community Health and Clinical Medicine for the Pacific* 1999; **6**(2): 211-2.

84. De Leo D, Milner A, Fleischmann A, et al. The WHO START study: suicidal behaviors across different areas of the world. *Crisis* 2013; **34**(3): 156-63.

85. Telisinghe PU, Colombage SM. Patterns of suicide in Brunei Darussalam and comparison with neighbouring countries in South East Asia. *Journal of Forensic and Legal Medicine* 2014; **22**: 16-9.

86. Wang D, Wang YT, Wang XY. Suicide in three ethnic groups in Huhhot, Inner Mongolia. *Crisis* 1997; **18**(3): 112-4.

87. Lu J, Xiao Y, Xu X, Shi Q, Yang Y. The suicide rates in the Yunnan Province, a multi-ethnic province in Southwestern China. *International Journal of Psychiatry in Medicine* 2013; **45**(1): 83-96.

88. Ali NH, Zainun KA, Bahar N, et al. Pattern of suicides in 2009: Data from the National Suicide Registry Malaysia. *Asia-Pacific Psychiatry* 2014; **6**(2): 217-25.

89. Jollant F, Malafosse A, Docto R, Macdonald C. A pocket of very high suicide rates in a non-violent, egalitarian and cooperative population of South-East Asia. *Psychological Medicine* 2014; **44**(11): 2323-9.

90. Hsieh SF, Liu BH, Pan BJ, Chang SJ, Ko YC. Mortality patterns of Taiwan Aborigines due to accidents. *Kaohsiung Journal of Medical Sciences* 1994; **10**(7): 367-78.

91. Cheng TA, Hsu M. A community study of mental disorders among four Aboriginal groups in Taiwan. *Psychological medicine* 1992; **22**(1): 255-63.

92. Wen CP, Tsai SP, Shih YT, Chung WSI. Bridging the gap in life expectancy of the Aborigines in Taiwan. *International Journal of Epidemiology* 2004; **33**(2): 320-7.

93. Liu IC, Liao SF, Lee WC, Kao CY, Jenkins R, Cheng ATA. A cross-ethnic comparison on incidence of suicide. *Psychological Medicine* 2011; **41**(6): 1213-21.

94. Machado DB, dos Santos DN. Suicide in Brazil, from 2000 to 2012. *Jornal Brasileiro de Psiquiatria* 2015; **64**(1): 45-54.

95. Orellana JD, Balieiro AA, Fonseca FR, Basta PC, Souza ML. Spatial-temporal trends and risk of suicide in Central Brazil: an ecological study contrasting Indigenous and non-Indigenous populations. *Revista Brasileira de Psiquiatria* 2016; **38**(3): 222-30.

96. Coloma C, Hoffman JS, Crosby A. Suicide among Guarani Kaiowa and Nandeva youth in Mato Grosso do Sul, Brazil. *Archives of Suicide Research* 2006; **10**(2): 191-207.

97. Souza MLP, Orellana JDY. Inequalities in suicide mortality between Indigenous and non-Indigenous people in the state of Amazonas, Brazil. *Jornal Brasileiro de Psiquiatria* 2013; **62**(4): 245-52.

98. Mao Y, Moloughney BW, Semenciw RM, Morrison HI. Indian reserve and registered Indian mortality in Canada. *Canadian Journal of Public Health* 1992; **83**(5): 350-3.

99. Isaacs S, Keogh S, Menard C, Hockin J. Suicide in the Northwest Territories: a descriptive review. *Chronic Diseases in Canada* 1998; **19**(4): 152-6.

100. Wotton K. Mortality of Labrador Innu and Inuit, 1971-1982. In: Fortuine R, editor. Circumpolar Health 84: Proceedings of the Sixth International Symposium on Circumpolar Health; 1985 May 13-18, 1984; Anchorage, Alaska: University of Washington Press; 1985. p. 139-42.

101. Hislop TG, Threlfall WJ, Gallagher RP, Band PR. Accidental and intentional violent deaths among British Columbia Native Indians. *Canadian Journal of Public Health* 1987; **78**(4): 271-4.

102. Macaulay A, Orr P, Macdonald S, et al. Mortality in the Kivalliq Region of Nunavut, 1987-1996. *International Journal of Circumpolar Health* 2004; **63 Suppl 2**: 80-5.

103. Ross CA, Davis B. Suicide and parasuicide in a northern Canadian Native community. *Canadian Journal of Psychiatry* 1986; **31**(4): 331-4.

104. Chandler MJ, Lalonde C. Cultural continuity as a hedge against suicide in Canada's First Nations. *Transcultural Psychiatry* 1998; **35**(2): 191-219.

105. Young TK. Mortality pattern of isolated Indians in northwestern Ontario: a 10-year review. *Public Health Reports* 1983; **98**(5): 467.

106. Pollock NJ, Mulay S, Valcour J, Jong M. Suicide rates in Aboriginal communities in Labrador, Canada. *American Journal of Public Health* 2016; **106**(7): 1309-15.

107. Fox J, Manitowabi D, Ward JA. An Indian community with a high suicide rate: 5 years after. *Canadian Journal of Psychiatry* 1984; **29**(5): 425-7.

108. Garro LC. Suicides by status Indians in Manitoba. *Arctic Medical Research* 1988; **47 Suppl 1**: 590-2.

109. Spaulding JM. Recent suicide rates among ten Ojibwa Indian bands in Northwestern Ontario. *Omega: Journal of Death and Dying* 1985; **16**(4): 347-54.

110. Mao Y, Morrison H, Semenciw R, Wigle D. Mortality on Canadian Indian reserves 1977-1982. *Canadian Journal of Public Health* 1986; **77**(4): 263-8.

111. Malchy B, Enns MW, Young TK, Cox BJ. Suicide among Manitoba's Aboriginal people, 1988 to 1994. *Canadian Medical Association Journal* 1997; **156**(8): 1133-8.

112. Penney C, Bobet E, Guimond E, Senécal S. Effect of community-level factors on suicide in Inuit Nunangat. *Canadian Diversity* 2009; **7**(3): 77-84.

113. Butler GC. Incidence of suicide among the ethnic groups of the Northwest Territories and Yukon Territory. *Medical Services Journal, Canada* 1965; **21**(4): 252-6.

114. Marshall D, Soule S. Accidental deaths and suicides among Alaska Natives, 1979-1994. *International Journal of Circumpolar Health* 1998; **57 Suppl 1**: 497-502.

115. Kraus RF, Buffler PA. Sociocultural stress and the American Native in Alaska: an analysis of changing patterns of psychiatric illness and alcohol abuse among Alaska Natives. *Culture, Medicine and Psychiatry* 1979; **3**(2): 111-51.

116. Kettl PA, Bixler EO. Suicide in Alaska Natives, 1979-1984. *Psychiatry* 1991; **54**(1): 55-63.

117. Holck P, Day GE, Provost E. Mortality trends among Alaska Native people: successes and challenges. *International Journal of Circumpolar Health* 2013; **72**.

118. Hlady WG, Middaugh JP. Suicides in Alaska: firearms and alcohol. *American Journal of Public Health* 1988; **78**(2): 179-80.

119. Forbes N, Van der Hyde V. Suicide in Alaska from 1978 to 1985: updated data from state files. *American Indian and Alaska Native Mental Health Research* 1988; **1**(3): 36-55.

120. Day GE, Provost E, Lanier AP. Alaska Native mortality rates and trends. *Public Health Reports* 2009; **124**(1): 54-64.

121. Day GE, Lanier AP. Alaska Native mortality, 1979-1998. *Public Health Reports* 2003; **118**(6): 518-30.

122. Andon HB. Patterns of injury mortality among Athabascan Indians in interior Alaska 1977-1987. *American Indian and Alaska Native Mental Health Research* 1997; **7**(3): 11-33.

123. Travis R. Suicide in Northwest Alaska. *White Cloud Journal of American Indian Mental Health* 1983; **3**(1): 23-30.

124. Travis R. Suicide and economic development among the Inupiat Eskimo. *White Cloud Journal of American Indian Mental Health* 1984; **3**(3): 14-21.

125. Wexler LM, Gone JP. Culturally responsive suicide prevention in indigenous communities: unexamined assumptions and new possibilities. *American Journal of Public Health* 2012; **102**(5): 800-6.

126. Lester D. Differences in the epidemiology of suicide in Asian Americans by nation of origin. *Omega: Journal of Death and Dying* 1994; **29**(2): 89-93.

127. Howard G, Peace F, Howard VJ. The contributions of selected diseases to disparities in death rates and years of life lost for racial/ethnic minorities in the United States, 1999-2010. *Preventing Chronic Disease* 2014; **11**: E129.

128. Young TJ, French LA. Suicide and social status among Native Americans. *Psychological Reports* 1993; **73**(2): 461-2.

129. Ogden M, Spector MI, Hill Jr CA. Suicides and homicides among Indians. *Public Health Reports* 1970; **85**(1): 75-80.

130. Sievers ML, Cynamon MH, Bittker TE. Intentional isoniazid overdosage among southwestern American Indians. *American Journal of Psychiatry* 1975; **132**(6): 662-5.

131. Conrad RD, Kahn MW. An epidemiological study of suicide among the Papago Indians. *American Journal of Psychiatry* 1974; **131**(1): 69-72.

132. Copeland AR. Suicide among nonwhites. The Metro Dade county experience, 1982-1986. *American Journal of Forensic Medicine and Pathology* 1989; **10**(1): 10-3.

133. Sievers ML, Nelson RG, Bennett PH. Adverse mortality experience of a southwestern American Indian community: overall death rates and underlying causes of death in Pima Indians. *Journal of Clinical Epidemiology* 1990; **43**(11): 1231-42.

134. Kalish RA. Suicide: An ethnic comparison in Hawaii. *Bulletin of Suicidology* 1968.

135. Herne MA, Bartholomew ML, Weahkee RL. Suicide mortality among American Indians and Alaska Natives, 1999-2009. *American Journal of Public Health* 2014; (S3): S336-42.

136. Lester D. Social correlates of American Indian suicide and homicide rates. *American Indian and Alaska Native Mental Health Research* 1995; **6**(3): 46-55.

137. Broudy DW, May PA. Demographic and epidemiologic transition among the Navajo Indians. *Social Biology* 1983; **30**(1): 1-16.

138. Becker TM, Samet JM, Wiggins CL, Key CR. Violent death in the West: Suicide and homicide in New Mexico, 1958-1987. *Suicide and Life-Threatening Behavior* 1990; **20**(4): 324-34.

139. Levy J. Navajo suicide. *Human Organization* 1965; **24**(4): 308-18.

140. Martin SL, Proescholdbell S, Norwood T, Kupper LL. Suicide and homicide in North Carolina: initial findings from the North Carolina Violent Death Reporting System, 2004-2007. *North Carolina Medical Journal* 2010; **71**(6): 519-25.

141. Humphrey JA, Kupferer HJ. Homicide and suicide among the Cherokee and Lumbee Indians of North Carolina. *Interational Journal of Social Psychiatry* 1982; **28**(2): 121-8.

142. Simpson SG, Reid R, Baker SP, Teret S. Injuries among the Hopi Indians: a population-based survey. *Journal of the American Medical Association* 1983; **249**(14): 1873-6.

143. Levy JE, Kunitz SJ. A suicide prevention program for Hopi youth. *Social Science & Medicine* 1987; **25**(8): 931-40.

144. Shore JH. American Indian suicide: fact and fantasy. *Psychiatry* 1975; **38**(1): 86-91.

145. Christensen M, Kightlinger L. Premature mortality patterns among American Indians in South Dakota, 2000-2010. *American Journal of Preventive Medicine* 2013; **44**(5): 465-71.

146. Wissow LS, Walkup J, Barlow A, Reid R, Kane S. Cluster and regional influences on suicide in a Southwestern American Indian tribe. *Social Science & Medicine* 2001; **53**(9): 1115-24.

147. Miller M. Suicides on a Southwestern American Indian reservation. *White Cloud Journal of American Indian Mental Health* 1979; **1**(3): 14-8.

148. Mullany B, Barlow A, Goklish N, et al. Toward understanding suicide among youths: results from the White Mountain Apache tribally mandated suicide surveillance system, 2001-2006. *American Journal of Public Health* 2009; **99**(10): 1840-8.

149. Silviken A, Haldorsen T, Kvernmo S. Suicide among indigenous Sámi in Arctic Norway, 1970-1998. *European Journal of Epidemiology* 2006; **21**(9): 707-13.

150. Indigenous and Northern Affairs Canada. Indian Status. 2016. https://www.aadnc-aandc.gc.ca/eng/ 1100100032374/1100100032378 (accessed October 25, 2017).
